# Genes Required for Aerial Growth, Cell Division, and Chromosome Segregation Are Targets of WhiA before Sporulation in *Streptomyces venezuelae*

**DOI:** 10.1128/mBio.00684-13

**Published:** 2013-09-24

**Authors:** Matthew J. Bush, Maureen J. Bibb, Govind Chandra, Kim C. Findlay, Mark J. Buttner

**Affiliations:** Department of Molecular Microbiology^a^; Department of Cell and Developmental Biology,^b^ John Innes Centre, Norwich, United Kingdom

## Abstract

WhiA is a highly unusual transcriptional regulator related to a family of eukaryotic homing endonucleases. WhiA is required for sporulation in the filamentous bacterium *Streptomyces*, but WhiA homologues of unknown function are also found throughout the Gram-positive bacteria. To better understand the role of WhiA in *Streptomyces* development and its function as a transcription factor, we identified the WhiA regulon through a combination of chromatin immunoprecipitation-sequencing (ChIP-seq) and microarray transcriptional profiling, exploiting a new model organism for the genus, *Streptomyces venezuelae*, which sporulates in liquid culture. The regulon encompasses ~240 transcription units, and WhiA appears to function almost equally as an activator and as a repressor. Bioinformatic analysis of the upstream regions of the complete regulon, combined with DNase I footprinting, identified a short but highly conserved asymmetric sequence, GACAC, associated with the majority of WhiA targets. Construction of a null mutant showed that *whiA* is required for the initiation of sporulation septation and chromosome segregation in *S. venezuelae*, and several genes encoding key proteins of the *Streptomyces* cell division machinery, such as *ftsZ*, *ftsW*, and *ftsK*, were found to be directly activated by WhiA during development. Several other genes encoding proteins with important roles in development were also identified as WhiA targets, including the sporulation-specific sigma factor σ^WhiG^ and the diguanylate cyclase CdgB. Cell division is tightly coordinated with the orderly arrest of apical growth in the sporogenic cell, and *filP*, encoding a key component of the polarisome that directs apical growth, is a direct target for WhiA-mediated repression during sporulation.

## IMPORTANCE

Since the initial identification of the genetic loci required for *Streptomyces* development, all of the *bld* and *whi* developmental master regulators have been cloned and characterized, and significant progress has been made toward understanding the cell biological processes that drive morphogenesis. A major challenge now is to connect the cell biological processes and the developmental master regulators by dissecting the regulatory networks that link the two. Studies of these regulatory networks have been greatly facilitated by the recent introduction of *Streptomyces venezuelae* as a new model system for the genus, a species that sporulates in liquid culture. Taking advantage of *S. venezuelae*, we have characterized the regulon of genes directly under the control of one of these master regulators, WhiA. Our results implicate WhiA in the direct regulation of key steps in sporulation, including the cessation of aerial growth, the initiation of cell division, and chromosome segregation.

## Introduction

The filamentous, Gram-positive bacterium *Streptomyces* has a complex life cycle, culminating in the formation of specialized reproductive structures called aerial hyphae, which extend upwards from the substrate mycelium into the air. Subsequently, the orderly arrest of aerial growth is coordinated with a remarkable synchronous septation event with concomitant segregation of chromosomes that leads to the formation of long chains of 50 to 100 unigenomic spores (1–4).

Genetic analysis of differentiation in the classical model organism, *Streptomyces coelicolor*, defined two classes of developmental mutant, blocked at distinct stages of the life cycle. *bld* mutants are unable to form aerial hyphae and therefore lack the characteristic “fuzzy” appearance of the wild type (WT), exhibiting a shiny, “bald” phenotype (1, 3). In contrast, *whi* mutants are able to erect aerial hyphae but are unable to complete the life cycle to form mature chains of spores. Such mutants fail to synthesize the characteristic polyketide pigment associated with mature wild-type spores and therefore appear white on plates (1, 3).

The principal focus of this work is the WhiA protein. *S. coelicolor whiA* mutants fail to halt aerial growth or to initiate sporulation septation and the partitioning of chromosomes. Instead, they keep growing, forming extremely long, undivided aerial hyphae containing uncondensed DNA that appears continuous on staining (5, 6). This phenotype is indistinguishable from that of *whiB* mutants, leading to suggestions that WhiA and WhiB function together in the same regulatory pathway (5, 6).

WhiA is of great interest, not only because of its critical biological role in controlling the initiation of sporulation but also because of the highly unusual nature of the protein itself, first predicted bioinformatically (7) and dramatically confirmed when the structure of a WhiA homologue from *Thermotoga maritima* was determined (8). The N-terminal domain of WhiA, constituting two-thirds of the protein, is homologous to eukaryotic “LAGLIDADG” homing endonucleases, but this domain is degenerate in that it has lost key residues required for metal binding and catalysis, and it also displays an extensively altered DNA-binding surface compared with true homing endonucleases (7, 8). Instead, DNA binding in WhiA is mediated through a smaller, C-terminal helix-turn-helix (HTH) domain that is completely absent from classical homing endonucleases (7–9). The two domains are connected by a flexible linker, and the C-terminal HTH domain binds DNA site-specifically by itself (9). Thus, the function of the much larger N-terminal degenerate homing endonuclease domain remains unknown.

A further intriguing aspect of WhiA biology is that it is not confined to streptomycetes or even sporulating actinomycetes but is present in essentially all Gram-positive bacteria, including, therefore, many nonsporulating genera (for example, *Staphylococcus*). This observation, combined with its relationship with homing endonucleases, raises very interesting questions about the evolution of WhiA as a transcription factor (10) and about the reassortment of the target genes under its control in sporulating and nonsporulating bacteria.

The regulons of the *bld* gene regulators BldA, BldD, BldH (also called AdpA), and, most recently, σ^BldN^ have all been characterized and reveal much of the network that governs the switch from vegetative to aerial growth (11–16). Thus, for example, we now know that σ^BldN^ controls the expression of the chaplin and rodlin proteins, the principal components of the hydrophobic sheath that enables the aerial hyphae to escape surface tension and extend into the air (16). However, the *whi* gene regulatory network that controls the later stages of the *Streptomyces* life cycle remains largely uncharacterized. Consequently, our understanding of the regulation of processes such as the orderly cessation of aerial growth, the initiation of sporulation septation, and chromosome segregation remains incomplete.

In-depth studies of the regulatory networks that control sporulation in *Streptomyces* have been greatly facilitated by the introduction of *Streptomyces venezuelae* as a new model system for the genus (16). The classical model system, *S. coelicolor*, poses certain logistical problems when seeking to characterize the regulons of the Bld and Whi master regulators. *S. coelicolor* sporulates only on solid medium, and the reproductive structures undergoing morphogenesis constitute just ~5% of the total biomass, making the application of global techniques such as chromatin immunoprecipitation-sequencing (ChIP-seq) to development-specific transcription factors problematic. This study takes advantage of *S. venezuelae*, which, unlike *S. coelicolor*, sporulates synchronously in liquid culture to near-completion (17), greatly facilitating application of global techniques to the analysis of differentiation (16). We apply *in vivo* ChIP-seq and microarray transcriptional profiling to confirm the function of WhiA as a transcription factor and to characterize the regulon of genes directly under its control. These results implicate WhiA in the direct control of key steps in sporulation, including the cessation of aerial growth, the initiation of cell division, and chromosome segregation.

## RESULTS AND DISCUSSION

### WhiA is required for sporulation in *S. venezuelae*.

**FIG 1:**
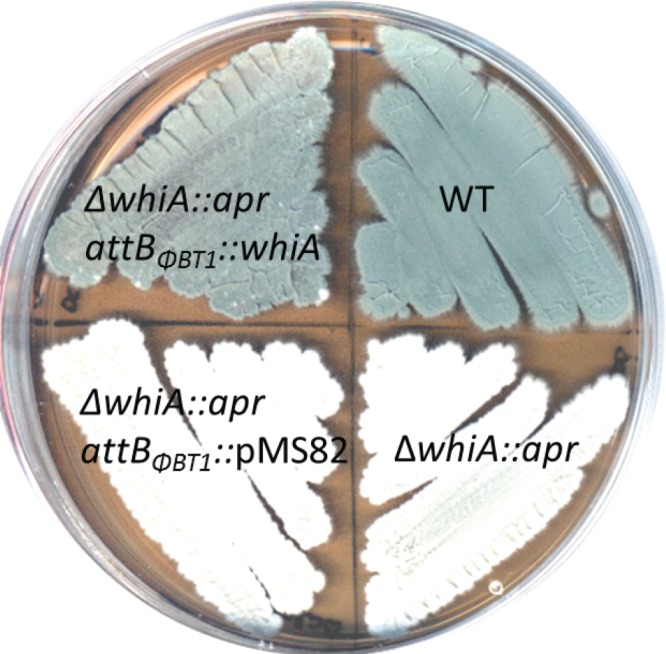
Phenotypes of wild-type *S. venezuelae* (WT), the constructed *ΔwhiA*::*apr* null mutant SV11 (Δ*whiA::apr*), SV11 carrying the empty vector pMS82 (*ΔwhiA*::*apr attB*_*ΦBT1*_::pMS82), and the complemented strain (Δ*whiA*::*apr attB*_*ΦBT1*_::*whiA*). Strains were grown on MYM solid medium and photographed after 4 days.

**FIG 2:**
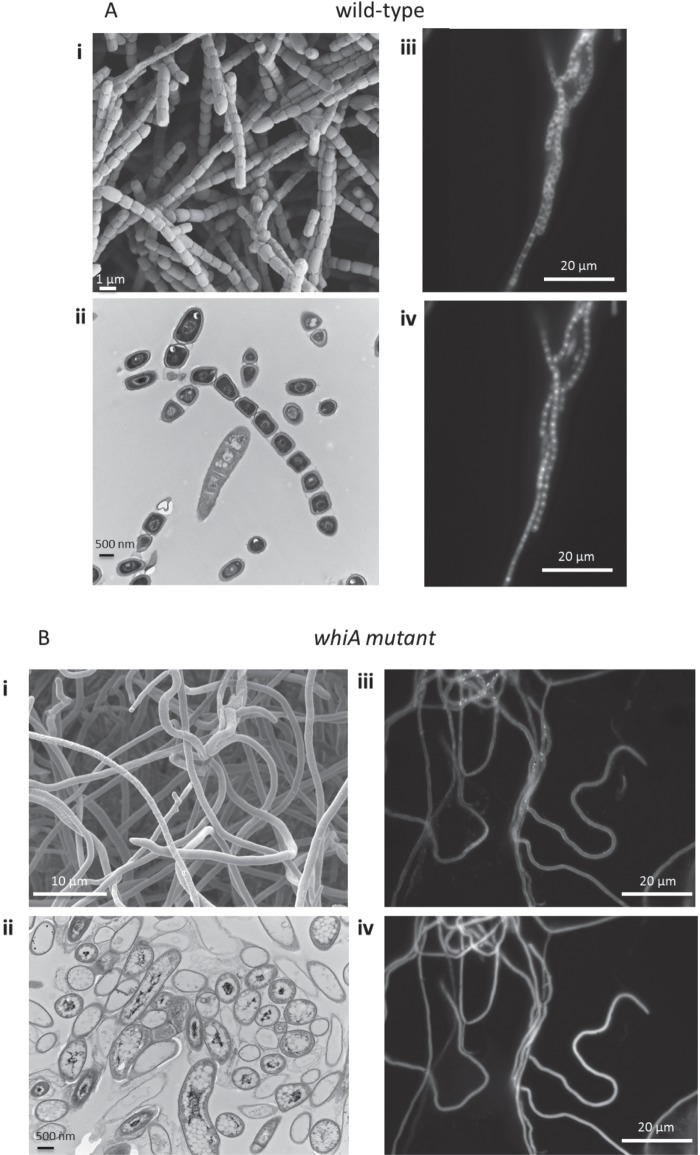
Phenotypes of wild-type *S. venezuelae* (A) and the constructed *ΔwhiA*::*apr* null mutant SV11 (B), examined by scanning electron microscopy (i), transmission electron microscopy (ii), and fluorescence microscopy after staining membranes and DNA with 10 µg/ml Nile red (iii) and 10 µg/ml DAPI (iv), respectively. Strains were grown on MYM solid medium for 2 days before fluorescence microscopy and 4 days before electron microscopy.

Before characterizing the WhiA regulon, a *whiA* null mutant of *S. venezuelae* was constructed to determine its phenotype and to compare it to the equivalent *S. coelicolor whiA* mutant (5). Using the Redirect PCR targeting method (18, 19), a *whiA* mutant was generated in which the coding region was replaced with an apramycin resistance (*apr*) cassette. As expected, the *S. venezuelae ΔwhiA*::*apr* strain was unable to sporulate on solid MYM (maltose-yeast extract-malt extract) medium or to synthesize the characteristic green spore pigment, instead appearing white (Fig. 1). In the wild type, the orderly arrest of aerial growth is followed by a septation event that divides the long, multigenomic hyphae into chains of unigenomic spores (Fig. 2A). To examine the effect of the *whiA* deletion in more detail, fluorescence microscopy of DAPI (4′,6-diamidino-2-phenylindole)-stained cells and scanning electron microscopy were conducted. As seen previously for *S. coelicolor whiA* mutants, the *S. venezuelae whiA* mutant formed unusually long aerial hyphae that fail to initiate septation or chromosome segregation (Fig. 2B). It should be noted that wild-type *S. venezuelae* forms straight aerial hyphae, in contrast to the coiled aerial hyphae made by wild-type *S. coelicolor*, and this is reflected in the phenotypes of the corresponding *whiA* mutants (Fig. 2B) (5, 6). All aspects of the *S. venezuelae whiA* null mutant were fully complemented in *trans* by a wild-type copy of *whiA* introduced into the ΦBT1 integration site (Fig. 1 and data not shown). Based on the phenotypes of the *whiA* mutants, we concluded that WhiA is likely to play similar roles in *S. venezuelae* and *S. coelicolor*.

### Defining the WhiA regulon.

In order to determine which genes are directly regulated by WhiA in *S. venezuelae*, ChIP-seq experiments were conducted. We constructed a strain of *S. venezuelae* that lacked *whiA* at its native locus but expressed an N-terminal, triple FLAG-tagged version of WhiA from the ΦBT1 integration site. This strain sporulated on solid MYM medium (see Fig. S1 in the supplemental material), although it displayed a slight delay in spore pigment production and was slower to sporulate in liquid culture. The best results were obtained when an additional [Gly_4_Ser]_3_ linker was engineered between the tag and WhiA. The resulting *S. venezuelae* strain sporulated to near-completion in liquid culture, albeit slightly slower than the wild type (Fig. S1 and data not shown). Furthermore, 3×FLAG-[Gly_4_Ser]_3_-WhiA was readily detected using anti-FLAG antibody in Western blot assays and there was no visible cross-reaction with any proteins in wild-type *S. venezuelae* (Fig. S2), suggesting optimal conditions for clean ChIP-seq experiments.

ChIP-seq was conducted with M2 anti-FLAG antibody at time points representing three distinct developmental stages: vegetative growth, the onset of sporulation (fragmentation), and midsporulation. The wild-type strain (expressing nontagged WhiA from the native locus) was used as a negative control to eliminate any signals that might arise from cross-reaction of the antibody with other DNA-binding proteins. In addition, total (nonimmunoprecipitated) input DNA from each time point was also subjected to sequencing. This very useful additional control enables nonuniform shearing of the chromosome to be taken into account. More relaxed regions of DNA shear more readily than compact regions, and so the former can give rise to higher background signals in ChIP-seq (20). Using *P* < 10^−4^ as the threshold for significance, a total of 284 peaks were detected in the FLAG-tagged WhiA strain over the three time points selected (see Fig. S3 and Table S1 in the supplemental material). Forty-eight of these were located more than 300 bp upstream of the nearest start codon (Table S1) and were not analyzed further. Using the same threshold for significance, only one peak was found in the wild-type (non-FLAG-tagged strain) control experiment. This peak (upstream of *sven5092*) also appeared in the FLAG-tagged WhiA strain and was subsequently removed from the data set.

Next, in order to determine how WhiA influences the expression of its target genes, wild-type *S. venezuelae* and the congenic *ΔwhiA* mutant were subjected to time-resolved, genome-wide transcriptional profiling during vegetative growth and sporulation. Strains were grown under the same conditions used for the ChIP-seq experiments. RNA samples were prepared at 2-hour intervals from 8 to 20 h, by which time sporulation was nearing completion, and following cDNA synthesis and labeling, samples were hybridized to Affymetrix DNA microarrays. Three independent biological replicates were performed for each strain, and the transcriptional profiling data were examined for each of the genes potentially under WhiA control. Almost all the peaks identified by ChIP-seq were found in intergenic regions. Of these, 40% were found in intergenic regions between divergent genes. In the case of these divergent regulatory regions, the transcriptional profiling data set was used to assess which gene was a target for regulation by WhiA.

Strikingly, 40% of WhiA target genes are downregulated in a *whiA* mutant and 35% are upregulated in a *whiA* mutant (see Table S1 in the supplemental material). This strongly suggests that WhiA is bifunctional, working almost equally as an activator and as a repressor to control differentiation in *S. venezuelae.*

### Identification of a WhiA consensus binding site.

**FIG 3:**
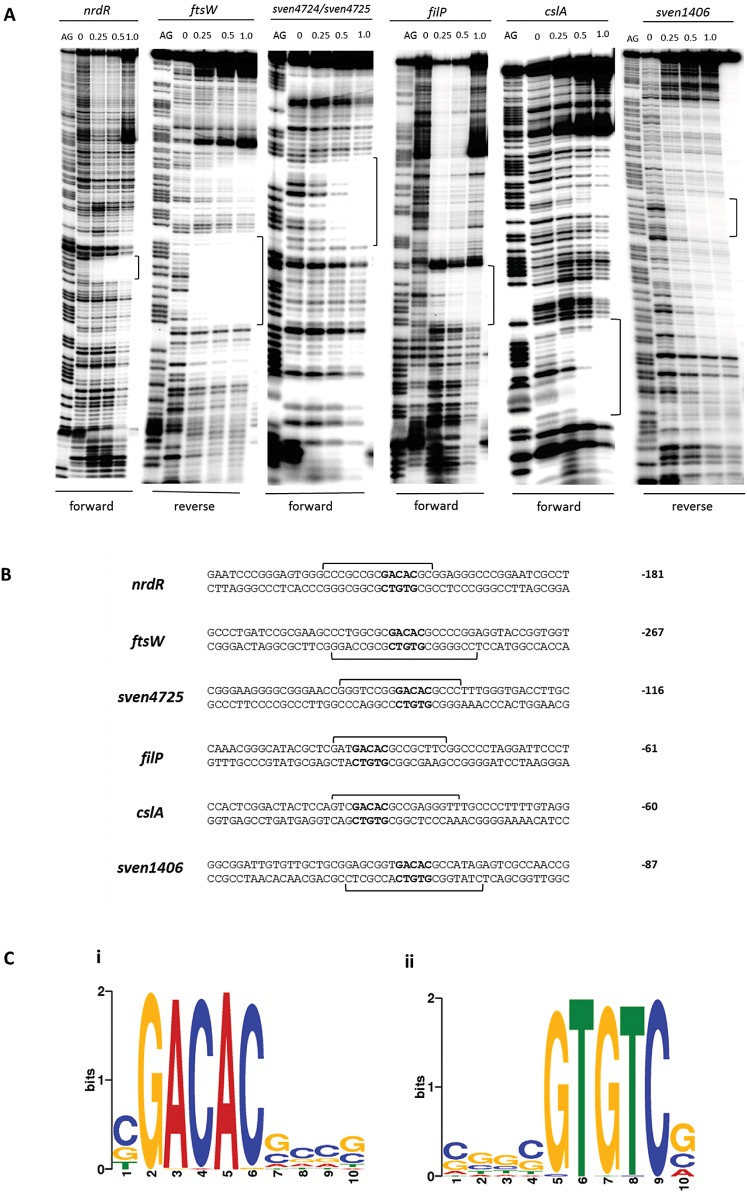
DNase I footprinting analysis of WhiA binding to the promoter regions of *nrdR*, *ftsW*, *filP*, *cslA*, and *sven1406* and the intergenic region between *sven4724* and *sven4725*. 5′-end-labeled probes were incubated with increasing concentrations of WhiA (indicated in μM above the lanes) and subjected to DNase I footprinting analysis as described in Materials and Methods. Footprints are flanked by Maxam and Gilbert sequence ladders (AG). Black brackets indicate the position of the WhiA-protected region. (B) Summary of DNase I footprinting results presented in panel A. Brackets indicate the protected sequences with the consensus GACAC motif shown in bold. The number indicates the distance (bp) between the consensus motif and the putative start codon of the downstream gene. (C) Overrepresented motif associated with ~70% of WhiA target genes, identified using the *MEME* algorithm (21). The height of the letters in the sequence logo, in bits, is proportional to the frequency of the A, C, T, or G nucleotides at each position of the motif. Both the forward (i) and the reverse complement (ii) versions of the consensus are shown.

To confirm and extend the ChIP-seq data and to help establish a consensus WhiA-binding sequence, DNase I footprinting analysis using purified N-terminally histidine-tagged WhiA was carried out on radiolabeled probes derived from the upstream regions of six of the strongest WhiA targets, as judged by ChIP-seq. In each case, addition of WhiA protein resulted in protection of a clearly defined region, consistent with high-affinity binding of monomeric WhiA (Fig. 3A). The sequences of these six confirmed WhiA target promoters were then examined using the *MEME* algorithm (21). This identified a short but perfectly conserved sequence (GACAC) in each of the submitted intergenic regions that correlated precisely with the position of the DNase I-protected region (Fig. 3B). This finding is in agreement with analysis conducted by Kaiser and Stoddard (9), in which WhiA binding was demonstrated at its own promoter. Following DNase I footprinting analysis, these authors tested the specificity of WhiA binding using a fluorescence competition assay and were able to determine those bases most important for the affinity of binding. Importantly, the GACAC motif independently identified here corresponds exactly with the sequence identified by Kaiser and Stoddard (9).

Following the identification of this short sequence, the remaining ChIP-seq targets were manually examined for the presence of the overrepresented WhiA-binding motif. Approximately 70% of the potential gene targets had a GACAC motif correlating closely with the position of the ChIP-seq peak. These target promoters were reexamined using the *MEME* algorithm, which failed to identify any additional overrepresented sequences associated with the GACAC motif (Fig. 3C). Within this subset, approximately 50% of genes showed greater than a 2-fold change in expression when comparing wild-type *S. venezuelae* to the *whiA* mutant. However, WhiA ChIP-seq targets that lacked a GACAC motif were just as likely to exhibit clear *whiA* dependence.

Because WhiA binds DNA as a monomer and it contains a large N-terminal homing endonuclease-like domain of unknown function, in addition to its small C-terminal HTH DNA-binding domain (8, 9), the orientation of the asymmetric GACAC motif relative to the target gene might well have regulatory significance. However, our analysis showed that there was no clear consistency in the directionality of the conserved motif, when present. We found that approximately twice the number of ChIP-seq-identified targets had upstream GACAC motifs compared to targets with the reverse complement GTGTC sequence upstream. Out of those ChIP-seq targets that show clear *whiA* dependence in microarray analysis, ~50% have upstream GACAC motifs, ~25% have upstream GTGTC motifs, and ~25% have no such sequence. Furthermore, this pattern does not change significantly when activated and repressed genes are analyzed separately. These data suggest that WhiA’s ability to function as an activator or as a repressor is not tied to its orientation on the DNA.

**FIG 4:**
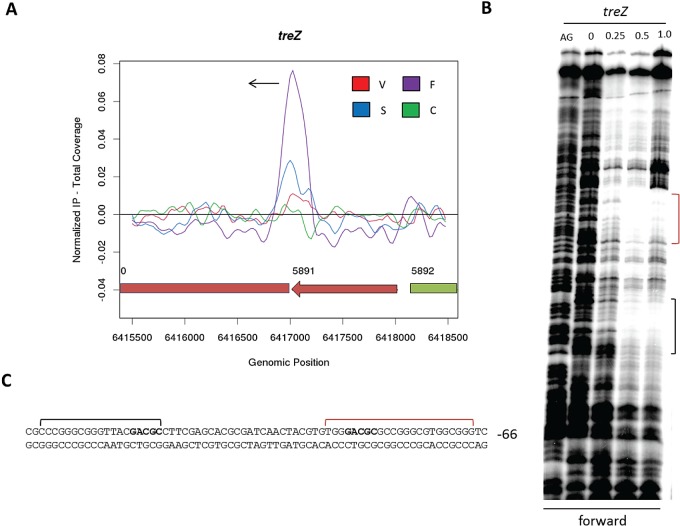
*treZ*, a WhiA target lacking a perfect GACAC motif. (A) ChIP-seq data for the WhiA target gene *treZ*. Color coding of the ChIP samples is as follows: 3×FLAG-[Gly_4_Ser]_3_-WhiA strain during vegetative growth (V, red), at the onset of sporulation/fragmentation (F, purple), and at midsporulation (S, blue) and the *S. venezuelae* negative-control strain (C, green). The plot spans approximately 3 kb of DNA sequence. Genes running left to right are shown in green, and genes running right to left are shown in red. The black arrow indicates the gene subject to WhiA regulation. (B) DNase I footprinting analysis of WhiA binding to the promoter region of *treZ*. 5′-end-labeled probes were incubated with increasing concentrations of WhiA (indicated in μM above the lanes) and subjected to DNase I footprinting analysis as described in Materials and Methods. The footprint is flanked by a Maxam and Gilbert sequence ladder (AG). Black and red brackets indicate the positions of the WhiA-protected regions. (C) Sequence summary of DNase I footprinting results presented in panel B. Black and red brackets indicate the protected sequences, with the near-consensus motif shown in bold. The number indicates the distance (bp) between the nearest consensus motif and the putative start codon of the *treZ* gene.

The identification of WhiA ChIP targets that depend on *whiA* for their expression, yet lack the GACAC motif, suggests that in some cases other sequences are sufficient to recruit WhiA. Three ChIP targets lacking the GACAC motif (*sven3535*, *sven3229*, and *treZ*, Table 1) were therefore tested for WhiA binding *in vitro* by DNase I footprinting. WhiA footprinted on only one of these three targets, *treZ* (*sven5890*). *treZ* is a clear WhiA target in ChIP-seq (Fig. 4A) that shows decreased expression in a *whiA* mutant, suggesting that WhiA activates transcription of this gene (see Table S1 in the supplemental material). DNase I footprinting of WhiA on the *treZ* promoter revealed two protected regions, both falling within the *in vivo* ChIP-seq peak (Fig. 4B). Although the determinants of this binding were not investigated, both protected regions contain the sequence GACGC, differing from the GACAC consensus sequence by 1 nucleotide (nt) (Fig. 4C). Thus, it is clear that WhiA can bind *in vivo* and *in vitro* to sites lacking the GACAC consensus sequence. For those WhiA ChIP-seq targets that did not bind WhiA *in vitro*, we speculate that additional transcription factors or accessory proteins might be required.

**TABLE 1:** Selected ChIP-identified WhiA targets

Flanking genes^*a*^		Distance^*b*^ (bp)	Product^*c*^	Adjusted *P* value^*d*^		
Left (−1)	Right (+1)			V	F	S
*ftsZ*		158	Cell division protein FtsZ	2.97E−04	2.42E−21	
	*ftsK*	11	Cell division protein FtsK		1.98E−18	
	*sepF3*	22	Cell division protein SepF3		1.50E−31	
*ftsW*		269	Cell division protein FtsW		1.01E−06	6.49E−05
*filP*		58	Coiled-coil protein FilP	2.75E−32	1.89E−39	7.12E−55
	*cslA*	54	Putative cellulose synthase CslA	8.68E−29	5.97E−51	1.41E−43
	*whiG*	298	RNA polymerase sigma factor WhiG		4.23E−16	
*pyrG*	*sven1411*	203; 447	CTP synthase; hypothetical protein		2.49E−72	7.72E−49
*sven4033*	*cdgB*	271; 55	Putative reductase; diguanylate cyclase CdgB		2.55E−06	
	*glpF*	130	Glycerol uptake facilitator protein GlpF		1.64E−10	
*sven3676*	*sven3677*	130; 649	Transcriptional regulator, PadR family; putative secreted penicillin binding protein		1.73E−13	
*sven4755*	*sven4756*	170; 79	Hypothetical protein; putative lipoprotein		5.48E−04	
*sven3340*	*crp*	−73; 259	Putative endonuclease; cyclic AMP-binding protein Crp		7.76E−07	5.18E−12
*sven3229*		14	Hypothetical protein		4.65E−10	
*sven3534*	*sven3535*	21; 167	Putative ABC transporter ATP-binding component; hypothetical protein	1.34E−09	7.88E−58	5.77E−50
*treZ*		47	Malto-oligosyltrehalose trehalohydrolase TreZ		1.54E−17	
*whiA*		−65	Sporulation transcription regulator WhiA		4.50E−07	
	*slzA*	133	Putative coiled-coil protein SlzA		3.73E−14	
*ftsE*	*sven2728*	175; 61	Cell division transporter, ATP-binding protein FtsE; putative membrane protein		1.33E−08	2.43E−10
*sven1406*		91	Chromosome (plasmid) partitioning ParA-like protein			9.45E−23
*sven5480*	*nrdR*	−76; 94	Hypothetical protein; ribonucleotide reductase transcriptional regulator NrdR	1.21E−06	5.11E−05	1.32E−21
*pyrR*	*bldD*	78; 169	Pyrimidine operon regulatory protein PyrR; regulator of morphogenesis and antibiotic production BldD	6.98E−04	5.04E−12	4.66E−15
*sigN*		271	RNA polymerase sigma factor SigN		3.97E−20	1.05E−10
*sven6396*	*wblH*	132; 63	Transcriptional regulator, IclR family; WhiB-type transcriptional regulator WblH		1.67E−27	4.98E−08
	*infA*	173	Translation initiation factor 1	6.70E−27	5.30E−65	2.25E−48
*efp*		191	Translation elongation factor P			5.64E−11
	*rpsH*	139	SSU ribosomal protein S8p		8.25E−14	1.99E−16
	*rpsJ*	75	SSU ribosomal protein S10p	4.50E−11		8.03E−06
*rpsA*	*sven1624*	132; 216	SSU ribosomal protein S1p; hypothetical protein			1.03E−06

^a^ Genes flanking the ChIP peak are listed.

^b^ Distance to the predicted start codon of the flanking genes.

^c^ (Possible) gene function based on annotation in StrepDB (http://strepdb.streptomyces.org.uk).

^d^ Maximum significance values (*P* < E−04) for the 3 ChIP samples; V, vegetative growth; F, onset of sporulation/fragmentation; S, midsporulation.

In Fig. 3, additional sites of weak protection were observed in the DNase I footprints on *nrdR*, *ftsW*, *cslA*, and the *parA*-like gene *sven1406*. It is not clear what these sites represent. Some contain GACAC motifs, and some contain the related sequence GACAG, but others do not show any obvious relationship to the consensus binding site.

### WhiA functions as an autoregulator.

**FIG 5:**
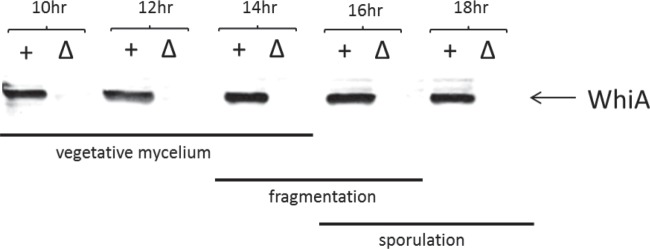
Western blot analysis of WhiA abundance during submerged sporulation in wild-type *S. venezuelae* (+) and in the congenic *whiA* mutant control strain, SV11 (Δ). Strains were grown in MYM liquid sporulation medium, 10 µg total protein was loaded per lane, and the blot was developed using a polyclonal antiserum raised against *S. venezuelae* WhiA. Bars indicate vegetative growth, fragmentation, and sporulation in the wild type, as judged by microscopic examination.

**FIG 6:**
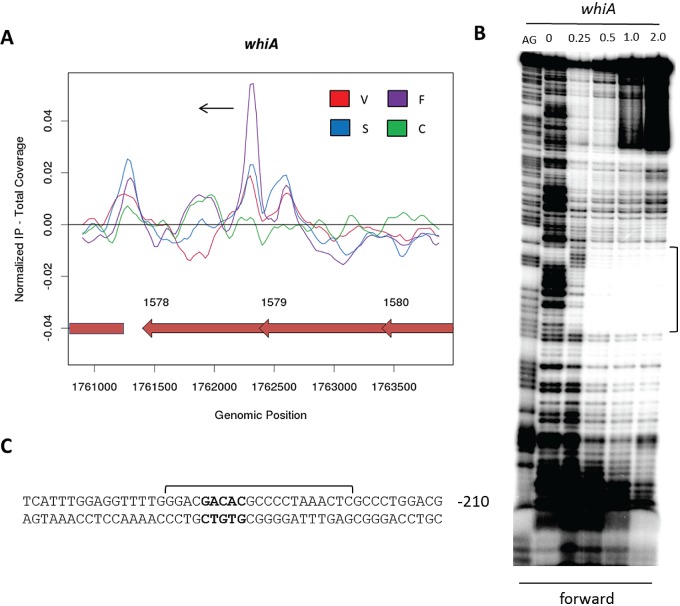
WhiA functions as an autoregulator. (A) ChIP-seq data for WhiA binding to the *whiAp2* promoter. Color coding of the ChIP samples is as follows: 3×FLAG-[Gly_4_Ser]_3_-WhiA strain during vegetative growth (V, red), at the onset of sporulation/fragmentation (F, purple), and at midsporulation (S, blue) and the *S. venezuelae* negative-control strain (C, green). The plot spans approximately 3 kb of DNA sequence. The black arrow indicates the gene subject to WhiA regulation. (B) DNase I footprinting analysis of WhiA binding to the *whiAp2* promoter. 5′-end-labeled probes were incubated with increasing concentrations of WhiA (indicated in μM above the lanes) and subjected to DNase I footprinting analysis as described in Materials and Methods. The footprint is flanked by a Maxam and Gilbert sequence ladder (AG). The black bracket indicates the position of the WhiA-protected region. (C) Sequence summary of DNase I footprinting results presented in panel B. The bracket indicates the protected sequence, and the consensus motif in shown in bold. The number indicates the distance (bp) between the consensus motif and the putative start codon of the *whiA* gene.

*whiA* appears to be the last gene in a four-gene operon, and previous work in *S. coelicolor* had suggested that *whiA* is transcribed throughout growth from a constitutive promoter located well upstream of the *whiA* gene itself (5). Consistent with this suggestion, our microarray transcriptional profiling data showed that *S. venezuelae whiA* is transcribed throughout vegetative growth and sporulation, and Western blot analysis of WhiA expressed from its native locus using polyclonal WhiA antiserum showed that the protein is similarly present throughout vegetative growth and sporulation (Fig. 5). In *S. coelicolor*, *whiA* expression is upregulated during development from a sporulation-specific promoter, *whiAp2*, lying just inside the upstream gene (5), and expression from *whiAp2* is reduced to a low constitutive level in a *whiA* null mutant, suggesting positive autoregulation (22). Furthermore, the ability of WhiA to bind to the *S. coelicolor whiAp2* promoter has been demonstrated *in vitro* (9). In line with these findings, our ChIP-seq experiments showed that *whiA* is a direct *in vivo* target of WhiA in *S. venezuelae* (Fig. 6A and Table 1). DNase I footprinting using purified N-terminally histidine-tagged *S. venezuelae* WhiA showed that WhiA binds to the *S. venezuelae whiAp2* promoter region (Fig. 6B and C), in agreement with equivalent experiments carried out in *S. coelicolor* (9). Overall, it appears that WhiA binds to the region immediately upstream of the *whiA* gene *in vivo* to increase its expression prior to sporulation in *Streptomyces*. These and other experiments reported here suggest that WhiA activity is not a simple reflection of WhiA abundance, likely implying that WhiA activity is regulated posttranslationally (see “Conclusions”).

### WhiA targets involved in polar growth.

**FIG 7:**
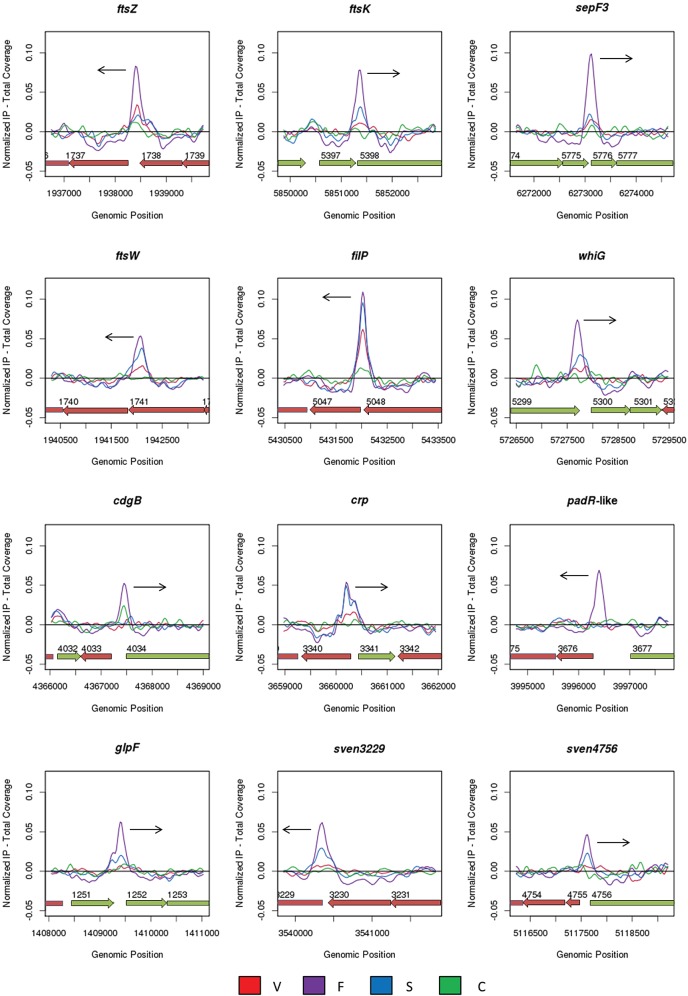
ChIP-seq data for 12 selected WhiA target genes: *ftsZ*, *ftsK*, *sepF3*, *ftsW*, *filP*, *whiG*, *cdgB*, *crp*, *sven3676*, *glpF*, *sven3229*, and *sven4756*. Color coding of the ChIP samples is as follows: 3×FLAG-[Gly_4_Ser]_3_-WhiA strain during vegetative growth (V, red), at the onset of sporulation/fragmentation (F, purple), and at midsporulation (S, blue) and the *S. venezuelae* negative-control strain (C, green). Plots span approximately 3 kb of DNA sequence. Genes running left to right are shown in green, and genes running right to left are shown in red. The black arrow indicates the gene subject to WhiA regulation. The arrangement of the 12 panels mirrors that in Fig. 8.

**FIG 8:**
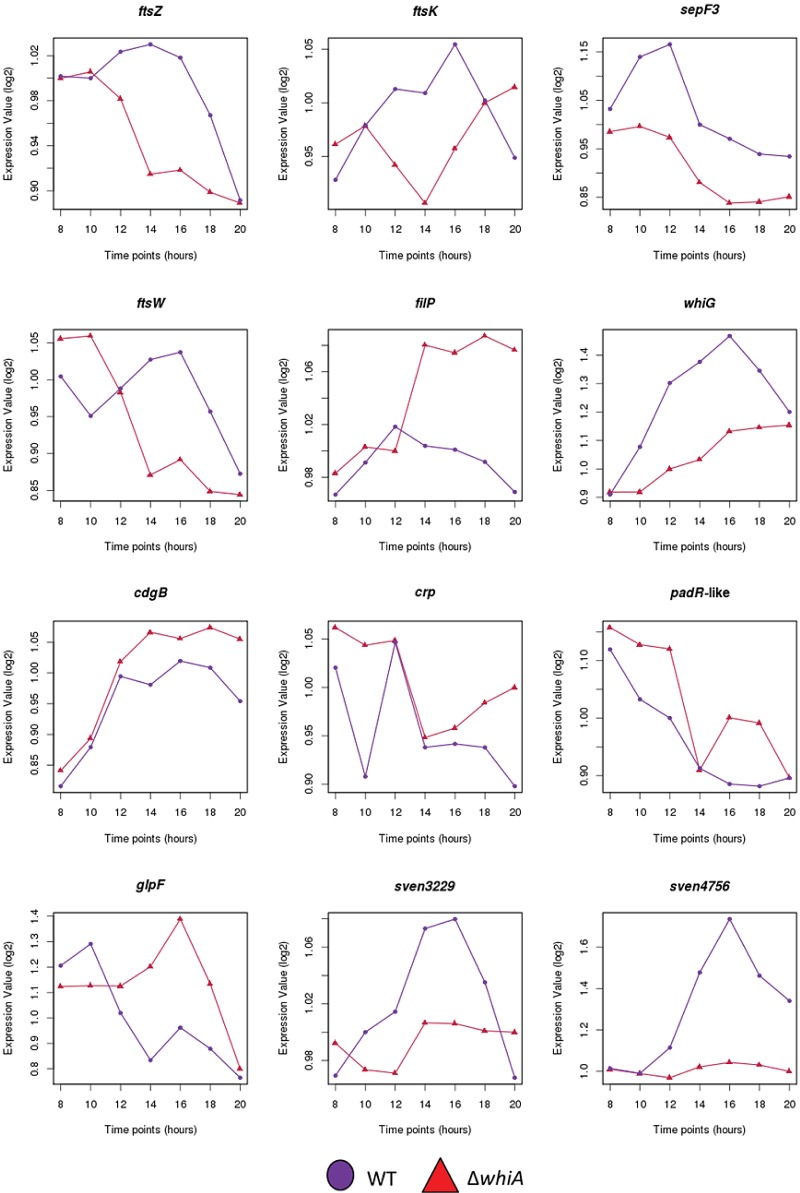
Microarray transcriptional profiling data for 12 selected WhiA target genes (*ftsZ*, *ftsK*, *sepF3*, *ftsW*, *filP*, *whiG*, *cdgB*, *crp*, *sven3676*, *glpF*, *sven3229*, and *sven4756*) during submerged sporulation in wild-type *S. venezuelae* (purple circles) and in the congenic *whiA* mutant, SV11 (red triangles). In each panel, the *x* axis indicates the age of the culture in hours, and the *y* axis indicates the per-gene normalized transcript abundance (log_2_). For the wild type, 10 to 14 h corresponds to vegetative growth, 14 to 16 h corresponds to the onset of sporulation (fragmentation), and 16 h onwards corresponds to sporulation. The arrangement of the 12 panels mirrors that in Fig. 7.

*Streptomyces* hyphae grow by tip extension, and this polar growth is directed by a complex of coiled-coil cytoskeletal proteins called the polarisome (also called the tip organizing center or TIPOC). The three known polarisome proteins are DivIVA, which is essential for viability of the organism, and two nonessential cytoskeletal proteins that nevertheless play vital roles in tip growth, Scy and FilP (23–26). All three members of the polarisome interact directly with each other, and Scy has been suggested to function as a molecular scaffold for the other components (26). The intermediate filament-like protein FilP is recruited by DivIVA and localizes just behind DivIVA at the hyphal tip (25). The reduced rigidity of the cell wall at hyphal tips in a *filP* mutant (revealed by atomic force microscopy) and its distorted morphology during growth suggest that FilP plays a mechanical role in controlling cell shape (27). Consistent with this suggestion, FilP can form potentially load-bearing net-like structures *in vitro* (25). These data suggest that FilP plays a true cytoskeletal role supporting tip growth. During differentiation, aerial hyphae arrest tip extension and initiate a massive, synchronous septation event, and during this switch the polarisome disappears (28–30). The orderly arrest of aerial growth requires *whiA* (5, 6), but the mechanisms underlying this dependence are unknown. One of the strongest WhiA targets, as judged by ChIP-seq, is *filP* (*sven5047*) (Fig. 7 and Table 1). Expression of *filP* is increased in a *whiA* mutant (Fig. 8), suggesting that WhiA represses *filP* transcription. Repression of *filP* by WhiA during development may therefore contribute to the orderly cessation of aerial growth prior to sporulation and thus help to explain why *whiA* mutants form abnormally long aerial hyphae. In the context of coiled-coil proteins like FilP, it is interesting that we also identified *slzA* (small leucine zipper protein A; *sven5271*) as a member of the WhiA regulon (Table 1). *slzA* encodes a coiled-coil protein of the leucine zipper class that interacts strongly with itself in bacterial two-hybrid assays, and *slzA* mutants show a mild developmental phenotype (A. Kotun and J. McCormick, personal communication).

Another WhiA target identified here is *cslA* (*sven5061*) (Table 1), which encodes a putative cellulose synthase that localizes to hyphal tips (31, 32). CslA is proposed to reinforce growing tips through cellulose production, and disruption of *cslA* leads to a severe delay in the formation of aerial hyphae (31, 32). Although the expression of *cslA* is largely unchanged in a *whiA* mutant, there is a 2-fold increase at a late stage in development (20 h), suggesting that WhiA represses *cslA* (see Table S1 in the supplemental material). *cslA* appears to be the first gene of a bicistronic operon that also includes *glxA*, encoding a galactose oxidase required for proper aerial development during osmotic stress. Although the two genes are cotranscribed, *glxA* is also expressed from an independent promoter within the *clsA* coding sequence (33).

### WhiA targets involved in cell division and chromosome segregation.

Strong signals for WhiA binding were found upstream of several key genes involved in cell division in *Streptomyces*, including *ftsZ*, *ftsW*, and *ftsK* (Fig. 7 and Table 1). Cell division is not essential for viability in *Streptomyces*, but it is required for sporulation, which involves the synchronous formation of a ladder of 50 or more cytokinetic rings (2). *ftsZ* transcription is under developmental control, being substantially upregulated from the *ftsZp2* promoter during sporulation (34). Further, in *S. coelicolor* at least, constitutive overexpression of *ftsZ* restores sporulation septation to *whi* mutants, suggesting that appropriate regulation of FtsZ levels is a vital function of the *whi* gene products (35). BldD was the first transcription factor to be identified as a direct regulator of *ftsZ* transcription, functioning to repress *ftsZ* expression during vegetative growth (14), when FtsZ is required only for the formation of occasional vegetative crosswalls, which divide hyphae but do not lead to constriction and cell-cell separation. Microarray transcriptional profiling conducted here shows that *ftsZ* expression is significantly downregulated in a *whiA* mutant, consistent with WhiA acting as a direct activator of *ftsZ* expression (Fig. 8).

Similarly, our data suggest that WhiA functions as an activator of *ftsW* and *ftsK* transcription via direct binding to their respective promoters (Fig. 7 and 8; Table 1). (Note that *ftsW* lies in the middle of the *dcw* operon, suggesting that the WhiA-binding site identifies an *ftsW*-specific promoter.) In *Escherichia coli*, FtsW is required to recruit its cognate transpeptidase, FtsI (PBP3), to the division site (36), and FtsW is also likely to function as the lipid II “flippase” (37), transporting bacterial cell wall precursors across the membrane to FtsI and other transpeptidases for incorporation into newly synthesized septal peptidoglycan. *S. coelicolor ftsW* null mutants grown under most conditions have a white phenotype and make aerial hyphae that are unable to assemble FtsZ rings, form sporulation septa, or segregate their chromosomes (38). However, cell division occurs independently of *ftsW* and *ftsI* when *S. coelicolor* is grown on low-osmolarity media (39). Since the *S. coelicolor* genome encodes four SEDS (shape, elongation, division, and sporulation)/penicillin binding protein (PBP) pairs, it is likely that another pair is able to support cell division under certain growth conditions. Indeed, FtsZ is the only known component of the division machinery absolutely required for cell division under all conditions in *Streptomyces*, although it is not essential for viability (2, 40).

Sporulation septa constrict over unsegregated nucleoids, and FtsK functions as a DNA translocase, removing trapped DNA from the closing septa (1–3, 41). FtsK localizes to sporulation septa, but it is not required for Z-ring assembly or sporulation septation. However, *ftsK* mutants have irregular DNA content in their spores and show greatly increased genetic instability, most often associated with large deletions at the ends of the linear chromosome, observations consistent with FtsK’s role as a septal DNA pump (41).

Our ChIP-seq data also reveal binding of WhiA upstream of the *ftsE* gene (Table 1). In *E. coli*, FtsE is required for constriction; it resembles the ATP-binding cassette of an ABC transporter, and it interacts with its membrane component, FtsX, as well as with FtsZ (42–45). Despite the presence of a WhiA ChIP peak upstream of the gene, *ftsE* does not show strong dependence on *whiA* for its expression (see Table S1 in the supplemental material).

Chromosome segregation during sporulation also requires the ParAB system. ParA is a Walker A ATPase that assists ParB in the formation of foci formed through the binding of ParB to ~20 *parS* sites that surround *oriC* (4, 46, 47). The observation that ParA forms filaments that originate from the tip and extend down the sporogenic cell suggests that it provides the force to organize the ParB-bound chromosomes prior to septation (4, 48). More recently, this process has been shown to be coordinated by a direct interaction between ParA and Scy, one of the components of the polarisome that directs tip extension in vegetative and aerial hyphae (30). A previous study showed that the sporulation-specific upregulation of *parAB* transcription in *S. coelicolor* depends, directly or indirectly, on several of the *whi* genes, including *whiA* (22), and *S. coelicolor* WhiA was shown to bind to the *parAB* promoter *in vitro* (9). Our microarray transcriptional profiling shows that the sporulation-specific upregulation of *parAB* transcription also depends on *whiA* and several other *whi* genes in *S. venezuelae*, but we did not detect binding of WhiA to the *parAB* promoter in our ChIP-seq experiments at any of the three time points examined. *sven1406* is annotated as *parA*-like, although its function has yet to be investigated. Our experiments identify *sven1406* as a direct WhiA target and show that its expression depends on *whiA* (Table 1; see also Table S1 in the supplemental material).

SepF functions as a component of the cell division machinery in *Bacillus*, where it is suggested to play an early role in the assembly of the FtsZ ring (49), in addition to a later role in constriction (50). In *S. venezuelae*, there are three SepF-like proteins (SepF1, SepF2, and SepF3), but the individual (and presumably related) functions of these homologues are unknown. Our data show that the *sepF3* (*sven5776*) promoter is a direct target of WhiA (Fig. 7 and Table 1) and that expression of *sepF3* is significantly downregulated in a *whiA* mutant, suggesting that WhiA activates transcription of *sepF3* (Fig. 8).

Overall, these results suggest that WhiA plays a central role in the initiation of sporulation in *Streptomyces* through the activation of multiple components of the cell division machinery.

### Other WhiA targets involved in morphological differentiation.

*cdgB* is a target that appears to be moderately repressed by WhiA during the later stages of differentiation (Fig. 7 and 8; Table 1). *cdgB* encodes a diguanylate cyclase that synthesizes c-di-GMP, and both overexpression and deletion of *cdgB* inhibit the formation of aerial hyphae (51), the first experiments to establish a role for c-di-GMP signaling in *Streptomyces* development. Strikingly, in *S. coelicolor*, BldD directly regulates *cdgB*, in addition to two other genes encoding predicted diguanylate cyclases, *cdgA* and *sco5511* (14, 51). The fact that *cdgB* is controlled by two key developmental regulators, WhiA and BldD, reinforces the emerging notion that c-di-GMP signaling plays an important role in the regulatory network controlling differentiation in *Streptomyces* (14, 51, 52).

**FIG 9:**
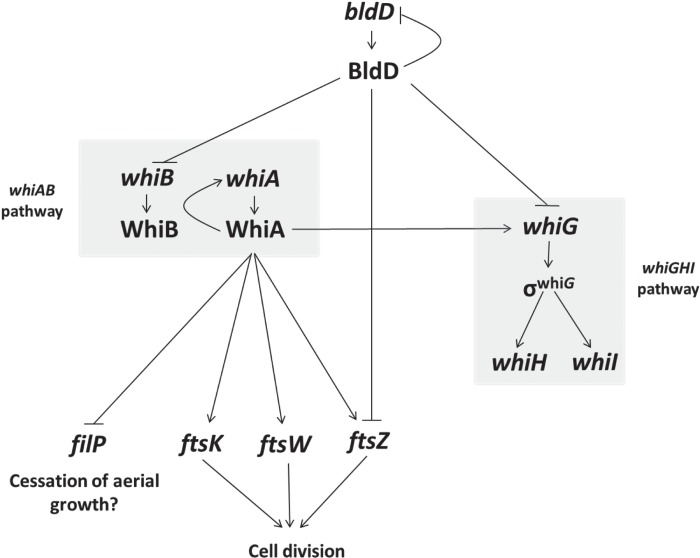
Model of the *whi* gene regulatory network. Flat-headed arrows indicate repression, and pointed arrows indicate activation.

ChIP-seq identified *whiG* (*sven5300*), encoding the sporulation-specific sigma factor σ^WhiG^, as a WhiA target (Fig. 7 and Table 1). Although the full σ^WhiG^ regulon has not been defined, σ^WhiG^ has been shown to directly activate transcription of *whiH* and *whiI*, encoding two further key sporulation transcription factors (Fig. 9) (53, 54). Our transcriptional profiling shows decreased *whiG* expression in an *S. venezuelae whiA* mutant, suggesting that WhiA activates *whiG* transcription (Fig. 8). Previous ChIP-seq studies showed that *whiG* is a target for regulation by BldD (14). Therefore, both the release of BldD-mediated repression of *whiG* and WhiA-mediated activation appear to contribute to the regulation of *whiG* expression during the transition from aerial growth to sporulation (Fig. 9).

It has been suggested, based on *in vitro* transcription, that σ^WhiG^ directs transcription of the *p2* promoter just upstream of *whiA* (9). However, Aínsa et al. (5) failed to detect Eσ^WhiG^-directed *in vitro* transcription from the *whiAp2* promoter under conditions in which the same preparation of σ^WhiG^ holoenzyme actively transcribed the well-validated σ^WhiG^ target promoter, *whiIp*. Further, Aínsa et al. (5) found, using S1 nuclease protection assays, that the *whiAp2* promoter is active in an *S. coelicolor whiG* deletion mutant, thus ruling out the *in vivo* dependence of *whiAp2* on σ^WhiG^. It has also been suggested that WhiA and σ^WhiG^ directly interact and that WhiA might function as a σ^WhiG^-specific anti-sigma factor (9). We have been unable to detect interaction between *S. venezuelae* WhiA and σ^WhiG^ in an *E. coli* bacterial two-hybrid system. Resolution of these issues will require further experimental work.

In *Streptomyces*, the *nrdR* gene encodes a repressor of the *nrdAB* and *nrdJ* genes, which encode class Ia and class II ribonucleotide reductases, respectively (55–57). Ribonucleotide reductases catalyze the reduction of nucleoside diphosphates (NDPs) into deoxynucleoside diphosphates (dNDPs), which are then converted into deoxynucleoside triphosphates (dNTPs), the immediate biosynthetic precursors of DNA, and ribonucleotide reductases play a critical role in regulating the total rate of DNA synthesis *in vivo* (58). Visualization of active replisomes using enhanced green fluorescent protein (EGFP) fused to a subunit of DNA polymerase III (DnaN-EGFP) indicates that, during the development of aerial hyphae, DNA replication is dramatically upregulated in the long, unseptated sporogenic cell, generating the 50 to 100 chromosomes that will eventually segregate into spores (59). This substantial enhancement of DNA replication presumably necessitates an increased supply of dNTPs. Here we show that WhiA directly binds to a region upstream of the *nrdR* gene and that *nrdR* expression is significantly upregulated in a *whiA* mutant (Table 1; see also Table S1 in the supplemental material), suggesting that WhiA represses the transcription of *nrdR*. Decreased levels of NrdR would, in turn, lead to derepression of *nrdAB* and *nrdJ* transcription and increased levels of the ribonucleotide reductases, thereby enhancing the levels of the dNTPs required for DNA synthesis in the sporogenic cell. Consistent with this logic, our microarray analysis also reveals a corresponding indirect dependence of *nrdAB* and *nrdJ* expression on WhiA prior to sporulation.

The largest ChIP-seq peak identified in our analysis lies upstream of the *pyrG* gene, encoding CTP synthase (Table 1), an enzyme that generates CTP from UTP, ATP, and glutamine. The expression of this gene is downregulated in a *whiA* mutant, suggesting that WhiA activates *pyrG* (see Table S1 in the supplemental material). Furthermore, we also identified *pyrR* (*sven1088*) as a WhiA target in ChIP-seq, and transcriptional profiling suggests that WhiA represses transcription of *pyrR* (Table 1; see also Table S1). In other bacteria, PyrR blocks pyrimidine biosynthesis by binding to mRNA derived from the *pyr* operon in the presence of uridine nucleotides (60). *pyrG* is not part of the *pyr* operon and therefore not regulated by PyrR. Activation of *pyrG* expression by WhiA might lead to an increase in CTP levels, but this could feed DNA replication in sporogenic hyphae only if it were converted to dCTP via three steps (CTP to CDP to dCDP to dCTP), and, apart from the ribonucleotide reductases, no other genes involved in dNTP synthesis were identified as WhiA targets. A speculative alternative explanation for the increase in *pyrG* expression during *Streptomyces* morphogenesis might be drawn from the observation that, in several organisms, CTP synthases have the ability to form cytoskeletal-like filaments (reviewed in references 61 and 62). In *Caulobacter crescentus*, these CTP synthase filaments appear to have been coopted for a cytoskeletal role in determining cell shape, regulating the curvature of cells independently of the enzyme’s catalytic function (63). The mechanism by which the CTP synthase filaments regulate cell shape involves cross talk with another cytoskeletal element, the intermediate filament crescentin (63). It will be interesting to see whether the developmental regulation of *pyrG* by WhiA reflects a similar cytoskeletal role for CTP synthase in *Streptomyces*.

Other direct WhiA targets identified here that are linked to morphological development include four additional genes encoding transcription factors: the cyclic AMP receptor protein (Crp), a PadR-family transcriptional regulator (*sven3676*), the SigB-like RNA polymerase sigma factor SigN (*sven3785*), and WblH (*sven6397*) (Fig. 7 and Table 1). Crp appears to function principally as a regulator of secondary metabolism and antibiotic production in *S. coelicolor*, but several Crp targets are associated with morphogenesis (64). WhiA appears to repress transcription of *crp* both during vegetative growth and following differentiation (Fig. 8). The PadR-like regulator is likely to function as a repressor to influence the expression of its downstream genes in *S. coelicolor*, and both deletion and overexpression of the corresponding gene lead to a bald phenotype with sparse aerial hyphae (65). Transcriptional profiling in *S. venezuelae* reveals clear upregulation in a *whiA* mutant (Fig. 8), suggesting that WhiA represses transcription of the PadR-family repressor gene. σ^SigN^ activity appears to be restricted to the “subapical stem,” the cell lying immediately below the sporogenic cell in aerial hyphae, where it plays a poorly defined role in morphological development and the general stress response (66). Our ChIP-seq analysis shows that *sigN* is a WhiA target in *S. venezuelae*, although our transcriptomic data suggest that WhiA does not exert a very substantial effect on *sigN* expression (see Table S1 in the supplemental material). WblH is a member of the Wbl (WhiB-like) family of *Actinobacteria*-specific proteins that also includes WhiB and WhiD (67–69). Wbl proteins contain four perfectly conserved cysteine residues that bind an oxygen-sensitive, nitric oxide (NO)-sensitive [4Fe-4S] cluster (70–72). The biochemical role of Wbl proteins has been a matter of dispute (reviewed in reference 68), but recent evidence strongly suggests that Wbl proteins function as transcription factors (73). The regulation of σ^WhiG^, σ^SigN^, Crp, WblH, and other transcription factors by WhiA emphasizes the pleiotropic potential of WhiA control.

*treZ*, the WhiA target investigated above that lacks the GACAC consensus sequence but carries two copies of a GACGC motif (Fig. 4), encodes the metabolic enzyme TreZ, which functions in a pathway that breaks down the storage compound glycogen to yield trehalose (74). The accumulation and degradation of glycogen, stored as insoluble granules, are under developmental control in *Streptomyces*, and glycogen reserves are actively degraded during sporulation (75, 76), leading to an accumulation of trehalose that contributes substantially to the heat and desiccation resistance of mature spores (77). Thus, the activation of *treZ* transcription by WhiA (see Table S1 in the supplemental material) provides a direct link between the regulatory cascade that controls differentiation in *Streptomyces* and the accumulation of trehalose in spores.

### WhiA targets involved in translation.

Unexpectedly, several of the WhiA targets that we identified through ChIP-seq are involved in general translation, including the genes encoding translation initiation factor 1 (IF-1), translation elongation factor P, small-subunit (SSU) ribosomal protein S8, SSU ribosomal protein S1, and SSU ribosomal protein S10 (Table 1; see also Table S1 in the supplemental material). Expression of each of these targets is significantly downregulated in a *whiA* mutant, suggesting that an increased abundance of these components of the translation machinery may be a feature of morphological development in *Streptomyces*. It should be noted that S1 is an RNA-binding protein that has also been coopted as a transcription termination factor (78), and S10 (NusE) is involved in antitermination in *E. coli* (79). It is therefore possible that these proteins are being used for something other than translation in *S. venezuelae*.

### Conclusions.

The work reported here confirms, on a genome-wide scale, that WhiA functions as a transcription factor and shows that it behaves almost equally as an activator and as a repressor. Further, supporting previous *in vitro* analysis (9), we show that most *in vivo* binding sites are associated with a highly conserved GACAC motif, but we find that there is no clear relationship between the orientation of this asymmetric sequence and whether the corresponding target gene is activated or repressed by WhiA.

WhiA consists of an N-terminal homing endonuclease domain and a C-terminal HTH domain, connected by a flexible linker (8), and the C-terminal HTH domain (which is completely absent from true homing endonucleases) binds DNA site-specifically by itself (9). It is therefore possible that the HTH domain of WhiA has entirely replaced the DNA-binding function of the homing endonuclease domain. However, although the homing endonuclease domain cannot footprint on DNA in isolation, it does appear to contribute to the affinity of DNA binding by full-length WhiA (9). Therefore, it is conceivable that the homing endonuclease domain could also contribute to the specificity of DNA binding. It may also mediate protein-protein interaction or be a target for posttranslational regulation controlling the activity of WhiA. Extensive further research will be required to understand how the evolutionarily adapted structural and biochemical features of WhiA enable it to fulfill its bifunctional role as repressor and activator.

Based on Western blot analysis, WhiA is constitutively present at all time points examined (Fig. 5; see also Fig. S2 in the supplemental material). In contrast, based on ChIP-seq analysis, WhiA binds to its target promoters predominantly at the onset of sporulation during fragmentation phase (Fig. 4, 6, and 7; see also Fig. S3). These results suggest that WhiA activity is subject to posttranslational regulation.

Counting both DNA strands, there are 15,116 GACAC sequences in the *S. venezuelae* chromosome, but only a fraction of these sequences are bound by WhiA *in vivo* (as judged by ChIP-seq). In addition, there are clear targets (such as *treZ*) that bind WhiA *in vivo* and *in vitro* but do not possess this exact motif. Additional specificity determinants must presumably exist in addition to the short (albeit highly conserved) GACAC motif. The shortness of this sequence and the consequent difficulties in bioinformatic prediction of true *in vivo* binding sites are reminiscent of eukaryotic transcription factors, something that could conceivably reflect the evolutionary history of WhiA.

Comparing the sequences of the *S. venezuelae* WhiA-binding sites defined by DNase I footprinting with the equivalent promoter regions in *S. coelicolor*, the GACAC motifs within the footprints are conserved in *S. coelicolor* for the *whiA*, *filP*, *ftsW*, *nrdR*, and *parA*-like targets but not for *cslA* and *sven4724/4725*. These results suggest that the WhiA regulon is likely to be broadly conserved between these two model species. However, because the GACAC sequence occurs so frequently in streptomycete genomes, it is not possible to make a valid global comparative analysis for WhiA-binding sites defined only by ChIP-seq.

**FIG 10:**
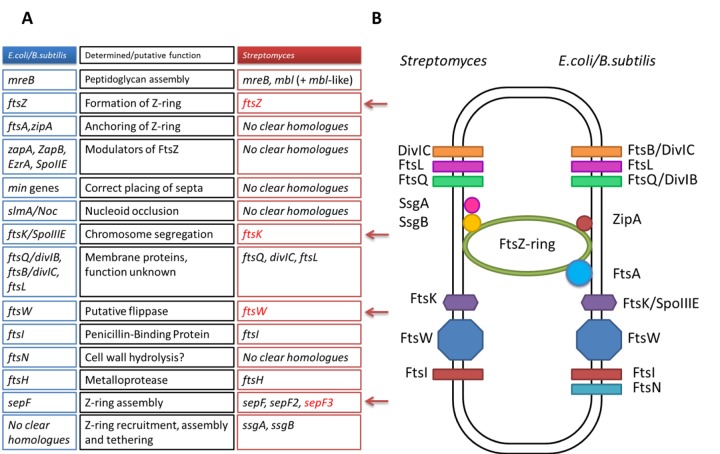
Comparison of the cell division machinery of *E. coli/B. subtilis* and *Streptomyces*. (A) Genes encoding key components of the divisome in *E. coli* and *B. subtilis*, their putative or determined function, and their presence or absence in *Streptomyces*. Components of the *Streptomyces* cell division machinery identified in this work as direct targets for WhiA regulation are highlighted in red and marked with an arrow. (B) Differences between the known components of the cell division machinery in *E. coli/B. subtilis* and *Streptomyces*.

Our constructed *S. venezuelae* null mutant shows that WhiA is required for the initiation of sporulation septation and chromosome segregation, as it is in *S. coelicolor*. It is therefore striking that several pivotal proteins of the *Streptomyces* cell division machinery, such as FtsZ, FtsW, and FtsK (Fig. 10), are encoded by genes that are direct targets for activation by WhiA during development. The initiation of the massive cell division event is tightly coordinated with the orderly arrest of apical growth in the sporogenic cell. It may therefore be significant that *filP* becomes repressed by WhiA at approximately the same time that *ftsZ*, *ftsW*, and *ftsK* are activated, given that FilP is a key component of the polarisome that directs polar growth at the tips of hyphae (25, 27). Further, recent evidence suggests that the direct interaction of another crucial component of the polarisome, Scy, with the partition protein ParA coordinates the arrest of tip extension in the sporogenic cell with the initiation of chromosome segregation prior to septation (30). The sporulation-specific upregulation of *parAB* transcription depends on *whiA* in *S. venezuelae*, but we did not detect binding of WhiA to the *parAB* promoter in ChIP-seq, suggesting that this dependence is indirect.

As illustrated in Fig. 10, many important components of the divisome in *E. coli* and *Bacillus subtilis* have no clear homologues in *Streptomyces*. For example, the FtsZ ring has to be anchored to the membrane, and in *E. coli*, this role is fulfilled by FtsA and ZipA (80), proteins absent in *Streptomyces* (2). This suggests that in *Streptomyces* there exist other proteins that perform functions central to the ability of the organism to divide and sporulate. The *Streptomyces* proteins SsgA and SsgB that function to recruit FtsZ during sporulation perfectly illustrate this point (81). Further cell division proteins unique to streptomycetes may well be under the control of one or more of the key developmental regulators, perhaps especially WhiA and WhiB, which are the only Whi regulators absolutely required for sporulation septation in *S. venezuelae* (M. J. Bibb and M. J. Buttner, unpublished data). In seeking out these proteins, it might be particularly fruitful to explore the overlap between the regulons of WhiA and WhiB, given their suggested close interplay and the near-identical phenotypes of the *whiA* and *whiB* mutants in both *S. coelicolor* and *S. venezuelae* (Fig. 9) (5, 6; Bibb and Buttner, unpublished).

WhiA-like proteins are found in virtually all Gram-positive bacteria, including many that do not sporulate, raising interesting questions about the evolution of WhiA and the reassortment of the genes under its control in different bacteria. Outside the streptomycetes, the only published genetic analysis of *whiA* function is in *Bacillus subtilis*. In this endospore-forming bacterium, deletion of *whiA* (*yvcL*) has no obvious phenotypic consequences (82), suggesting that WhiA does not play an important role in the control of sporulation or cell division in this organism. It will be interesting to confirm that WhiA functions as a transcription factor in *Bacillus* and, if so, to identify the genes it regulates.

## MATERIALS AND METHODS

### Bacterial strains, plasmids, oligonucleotides, and growth conditions.

Strains, plasmids, and oligonucleotides used in this study are described in Table S2 in the supplemental material. *Escherichia coli* K-12 strain DH5α was used for plasmid and cosmid propagation. BW25113 (83) containing a λ RED plasmid, pIJ790, was used to create the disruption cosmid. Cosmids were conjugated from the *dam dcm hsdS E. coli* strain ET12567 containing pUZ8002 (84) as described by Gust et al. (18, 19). Although ET12567 was used for this purpose, it should be noted that *S. venezuelae* does not restrict methylated DNA, and so cosmids can be conjugated from methylating *E. coli* strains. *S. venezuelae* strains were cultured in MYM-TAP (85) made with 50% tap water and supplemented with 200 µl of trace element solution (86) per 100 ml. Conjugations between *E. coli* and *S. venezuelae* were essentially carried out as described for *S. coelicolor* (86). However, because *S. venezuelae* grows much more quickly than *S. coelicolor*, the plates were incubated either at room temperature overnight or at 30°C for only 7 h before overlaying with the selective antibiotics (incubation at 30°C for longer periods before overlaying results in confluent lawns of *S. venezuelae*). Additionally, there is no need to heat shock *S. venezuelae* spores prior to the mating. Where conjugation was carried out using the nonsporulating *ΔwhiA*::*apr* strain, MYM liquid cultures were set up using mycelium as an inoculum and actively growing cultures were concentrated and used in the conjugation.

Spores of wild-type *S. venezuelae* were harvested from MYM plates using sterile cotton pads. Three milliliters of 20% glycerol was gently applied across the plate using sterile forceps and the cotton pad. The spores were collected through the pad using a 2-ml syringe. For the nonsporulating *whiA* mutant, mycelium in 20% glycerol was homogenized manually 10 times in a Dounce homogenizer and appropriate (and reproducible) inocula were determined in trial experiments, as judged by optical density (OD) and microscopic inspection.

### Construction and complementation of an *S. venezuelae whiA* null mutant.

Using the Redirect PCR targeting method of Gust et al. (18, 19), *whiA* mutants were generated in which the coding region was replaced with a single apramycin resistance (*apr*) cassette. A cosmid library that covers >98% of the *S. venezuelae* genome (Bibb and Buttner, unpublished) is fully documented at http://strepdb.streptomyces.org.uk/. Cosmid Sv-2-B11 was introduced into *E. coli* BW25113 containing pIJ790, and the relevant genes were replaced with the *apr-oriT* cassette amplified from pIJ773 using the primer pairs whiAdisfor and whiAdisrev (see Table S2 in the supplemental material). The resulting disrupted cosmids were confirmed by restriction digestion and by PCR analysis using the flanking primers whiAconfor and whiAconrev and introduced into *S. venezuelae* by conjugation. Null mutant derivatives, generated by double crossing-over, were identified by their apramycin-resistant, kanamycin-sensitive, and morphological phenotypes, and their chromosomal structures were confirmed by PCR analysis using the flanking primers whiAconfor and whiAconrev and by Southern hybridization using the entire cosmid Sv-2-B11, partially digested with Sau3AI, as a probe. A representative *whiA* null mutant was designated SV11. For complementation, *whiA* was amplified with the primers whiAcompfor and whiAcomprev, generating a fragment carrying the coding sequence and the *whiA* promoter, and cloned into HindIII-cut pMS82 (87) to create pIJ6760. The plasmid was introduced into the *whiA* mutant by conjugation and fully complemented all aspects of the mutant phenotype.

### Construction of a 3×FLAG-WhiA-complemented *S. venezuelae* strain.

In order to engineer an *S. venezuelae* strain expressing a form of WhiA with an N-terminal, triple-FLAG tag (DYKDHDGDYKDHDIDYKDDDDK), a pMS82-derived construct, pIJ10600, was created via a two-step fusion-PCR approach. In the first step, the Sv-2-B11 cosmid was used as a template for two separate PCRs. The first reaction amplified the promoter region of the *whiA* gene using the primer pair whiAFLAGextfor and whiAFLAGfusrev. The second reaction amplified the coding region of the *whiA* gene using the primer pair whiAFLAGfusfor and whiAFLAGextrev. Together, the whiAFLAGfusrev and whiAFLAGfusfor primers contain the sequence encoding the triple-FLAG tag via a 24-bp overlapping section. In the second step, a PCR using the nested primers whiAFLAGnesfor and WhiAFLAGnesrev was used to amplify the entire *whiA* gene and its promoter, fusing the two products from step 1 together and incorporating the 3×FLAG tag sequence between them. The nested primers whiAFLAGnesfor and WhiAFLAGnesrev additionally contain the HindIII and KpnI sites, respectively, to enable cloning into HindIII-, KpnI-cut pMS82. The resulting vector was named pIJ10600. In order to insert a [Gly_4_Ser]_3_ linker between the 3×FLAG tag and the coding region of *whiA*, a second fusion experiment was conducted using pIJ10600 as a template. Once again, two PCRs were carried out in a first step using the primer pairs whiAFLAGnesfor/whiALINKfusrev and whiAFLAGnesrev/whiALINKfusfor. Together, the whiALINKfusrev and whiALINKfusfor primers contain the sequence encoding the [Gly_4_Ser]_3_ linker (GGTGGAGGCGGTTCAGGCGGAGGTGGCTCTGGCGGTGGCGGTAGT) via a 24-bp overlapping section. The second step, employing the same nested primers, resulted in fusion of the two products in the first step and incorporated the linker sequence between them. Restriction digestion using HindIII and KpnI, followed by ligation into HindIII-, KpnI-cut pMS82, created pIJ10601. The plasmids pIJ10600 and pIJ10601 were introduced individually into the *ΔwhiA* mutant by conjugation, and their ability to restore sporulation was assessed both on solid and in liquid MYM.

### RNA isolation and DNA microarray analysis.

RNA isolation from *S. venezuelae* and DNA microarray analysis were performed as described previously (16, 88). The CEL files received from the scanner were read into the R package for statistical computing (89) using the *ReadAffy* function of the *affy* package (90). The *rma* function of the *affy* package was used to compute an *ExpressionSet* object from the CEL files. This *ExpressionSet* object contains the expression values (log_2_) for each gene in each CEL file. The function *lmFit* of the *limma* package (91), along with a suitable design matrix, was used to combine replicate arrays into single coefficients of expressions for each gene at each time point or strain into an *MArrayLM* object. Expression values were retrieved from the *MArrayLM* object and subjected to a per-gene normalization to the median before being used to generate the graphs shown in this paper.

### Chromatin immunoprecipitation.

*S. venezuelae* ATCC 10712 and *S. venezuelae* SV11-pIJ10601 (Δ*whiA*::*apr attB*_*ΦBT1*_::3×FLAG-[Gly_4_Ser]_3_-*whiA*) were grown in four 30-ml volumes of MYM-TAP for the appropriate length of time. Formaldehyde was added to cultures at a final concentration of 1% (vol/vol), and incubation was continued for 30 min. Glycine was then added to a final concentration of 125 mM to stop the cross-linking. The samples were left at room temperature for 5 min and washed twice in phosphate-buffered saline (PBS) buffer (pH 7.4). The pellets were resuspended in 0.5 ml of lysis buffer (10 mM Tris-Cl, pH 8.0, 50 mM NaCl), containing 15 mg ml^−1^ of lysozyme and 1× protease inhibitor (Roche Applied Science) and incubated at 37°C for 25 min or until lysed. Subsequently, 0.5 ml immunoprecipitation (IP) buffer (50 mM Tris-Cl, pH 8.0, 250 mM NaCl, 0.8% [vol/vol] Triton X-100), containing 1× protease inhibitor, was added, and the samples were chilled on ice. The samples were sonicated for six cycles of 20 s each at 10 µm to shear chromosomal DNA into fragments ranging from 300 to 1,000 bp on average. The samples were centrifuged twice at 16,000 × *g* at 4°C for 10 min to clear the cell extracts, after which 50 µl of each was set aside for total-DNA extraction. The remaining lysates were added to M2 (Sigma-Aldrich A2220) gel suspension (40 µl per ml of lysate), prepared and washed as described in the manufacturer’s instructions. The mixtures were then incubated on a rotating wheel at 4°C overnight. The samples were centrifuged for 30 s at 4°C and 5,500 × *g*, and the pellets were washed twice with 0.5× IP buffer and then twice with 1× IP buffer and transferred to new Eppendorf tubes after the first washing step. The pellets and 50 µl of total cell extracts (set aside earlier) were eluted overnight at 65°C in 100 µl of IP elution buffer (50 mM Tris-Cl, pH 7.6, 10 mM EDTA, 1% SDS) to reverse the cross-links. The samples were centrifuged at 16,000 × *g* for 5 min to remove the beads. The pellets were reextracted with 50 µl of TE buffer (10 mM Tris-Cl, pH 7.4, 1 mM EDTA) and incubated with 0.2 mg ml^−1^ proteinase K (Roche) for 2 h at 55°C. The samples were extracted twice with phenol-chloroform and once with chloroform and further purified using QiaQuick columns (Qiagen). DNA was eluted in 50 µl EB buffer (10 mM Tris-Cl, pH 8.5) and quantified using a NanoDrop spectrophotometer (Thermo Scientific).

### Library construction and sequencing.

Library construction and sequencing were performed by The Genome Analysis Centre (TGAC), Norwich Research Park, Norwich, United Kingdom. The TruSeq ChIP sample preparation kit from Illumina Inc. (part 15023092, revision A, August 2012) was used according to the recommended protocol. In brief, 5 to 10 ng ChIP DNA is blunt ended and phosphorylated, and a single “A” nucleotide is added to the 3′ ends of the fragments in preparation for ligation to an adapter with a single-base “T” overhang. The ligation products are purified and accurately size selected by agarose gel electrophoresis. Size-selected DNA is then purified and PCR amplified to enrich for fragments that have adapters on both ends. The final purified product is then quantified using a combination of Bioanalyzer DNA HS Chip from Agilent Technology 2100 Bioanalyzer and Qubit 2.0 from Invitrogen. The libraries were normalized to 10 nM and pooled, and quantitative PCR (q-PCR) was performed prior to cluster generation.

Each library pool was diluted to 2 nM with NaOH, and 5 µl was transferred into 995 µl HT-1 to give a final concentration of 10 pM. The normalized library (120 µl) was then transferred into a 200-µl strip tube and placed on ice before loading onto the Illumina cBot. Flow cells were clustered using TruSeq Single-Read Cluster Generation Kit v3, using the SR_Amp_Lin_Block_Hyb_v8.0 recipe. Following the clustering procedure, the flow cell was loaded onto the Illumina HiSeq2000 instrument according to the manufacturer’s instructions. The sequencing chemistry used was TruSeq SBS version 3 using Illumina software HCS 1.5.15.1 and RTA 1/13/48. Each library pool was run on the Illumina HiSeq2000 instrument with 100-bp single-read run metrics.

### ChIP-seq data analysis.

The reads in the fastq files received from the sequencing contractor were aligned to the *S. venezuelae* genome (GenBank accession number NC_018750) using the bowtie2 software (92), which resulted in one SAM (.sam) file for each fastq file. All further operations described below were carried out using a combination of Perl scripts dependent on the BioPerl toolkit (93) and R (89) scripts. From each SAM file, coverage at (number of reads mapping to) each nucleotide position of the *S. venezuelae* genome was calculated and the output was saved in files here referred to as coverage files. For each coverage file, a local enrichment was calculated in a moving window of 51 nucleotides (nt) moving in steps of 25 nucleotides as (the sum of coverage at each nucleotide position in the 51-nt window) divided by (the sum of coverage at each nucleotide position in a 2,001-nucleotide window centered around the 51-nucleotide window). This results in an enrichment ratio value for every 25 nucleotides along the genome. The enrichment ratios thus calculated were stored in files referred to as enrichment files.

In each difference file, the *P* value of each difference was calculated assuming that the differences were normally distributed using the *pnorm* function of R.

Now, for each pair of total DNA and IP DNA enrichment ratios, the values of total DNA were subtracted from the values of IP DNA and the differences stored in files here referred to as the difference files. Thus, we arrived at a difference file for each of the three time points for the FLAG-tagged pairs and one file for the wild-type (WT) pair. In each difference file, the *P* value of each difference was calculated assuming that the differences were normally distributed using the *pnorm* function of R. Differences with adjusted *P* values (using the *pnorm* function of R with the Hochberg method) less than or equal to 1e−4 were saved to new files referred to as sigDiff files. All the four types of files, i.e., coverage, enrichment, difference, and sigDiff, are two-column files where the first column is always position on the genome and the second column contains the relevant value.

The gene coordinates in the GenBank file and the genomic positions in the sigDiff files were combined to arrive at lists of genes on the left and right of the significantly different genomic positions. The relationships of the difference values and these genes were manually verified by viewing the difference files and the features in the *S. venezuelae* GenBank file as tracks in the Integrated Genome Browser (94). Significant different genomic positions located more than 300 bp upstream of the nearest start codon were separated from the data set. The most significant genomic position for each transcription unit was then selected. Finally, the information above was combined with Affymetrix microarray data to generate Table S1 in the supplemental material.

### Western blotting, DNase I footprinting, and microscopy. 

For details on Western blotting, DNase I footprinting, and microscopy, see Text S1 in the supplemental material.

## SUPPLEMENTAL MATERIAL

Text S1 Supplemental materials and methods. Download Text S1, DOCX file, 0.1 MB

Figure S1 Phenotypes of wild-type *S. venezuelae* (WT), the constructed *ΔwhiA*::*apr* null mutant SV11 (*ΔwhiA*::*apr*), SV11 carrying the empty vector pMS82 (*ΔwhiA*::*apr attB*_*ΦBT1*_::pMS82), and SV11 expressing WhiA (*ΔwhiA*::*apr attB*_*ΦBT1*_::*whiA*) or N-terminal, triple FLAG-tagged versions of WhiA with or without a [Gly_4_Ser]_3_ linker (*ΔwhiA*::*apr attB*_*ΦBT1*_::3×FLAGlink*-whiA* and *ΔwhiA*::*apr attB*_*ΦBT1*_::3×FLAG*-whiA*). (A) Appearance on MYM solid medium after 4 days. (B) Phase-contrast images of coverslip impressions taken from MYM plates after 4 days. Download Figure S1, EPS file, 4.4 MB

Figure S2 Western blot analysis of wild-type *S. venezuelae* (−) and *ΔwhiA*::*apr attB*_*ΦBT1*_::3×FLAG-[Gly_4_Ser]_3_-*whiA* (+) grown in MYM liquid cultures and sampled from 14 to 40 h. Five micrograms total protein was loaded per lane, and 3×FLAG-[Gly_4_Ser]_3_-WhiA was detected using M2 anti-FLAG antibody. Protein size markers (NEB) are shown with molecular masses in kDa. Download Figure S2, EPS file, 0.3 MB

Figure S3 Chromosome-wide distribution of WhiA-binding sites in *S. venezuelae* identified by ChIP-seq analysis. ChIP-seq was conducted using M2 anti-FLAG antibody on the *ΔwhiA*::*apr attB*_*ΦBT1*_::3×FLAG-[Gly_4_Ser]_3_-*whiA* strain at time points representing three distinct developmental stages: vegetative growth, the onset of sporulation (fragmentation), and midsporulation. The wild-type strain (expressing nontagged WhiA from the native locus) analyzed at the onset of sporulation (fragmentation) was used as a negative control. Download Figure S3, EPS file, 0.8 MB

Table S1 Complete ChIP-seq data set for *ΔwhiA*::*apr attB*_*ΦBT1*_::3×FLAG-[Gly_4_Ser]_3_-*whiA*. Each row represents a ChIP “peak” based on the analysis of 25-bp segments of the *S. venezuelae* genome. Only those peaks with significance of *P* < E−04 for at least one of the ChIP samples are included in the analysis. A single entry that had significance of <E−04 in the negative control was discarded from the analysis. Pos, position of peak in the *S. venezuelae* genome in bases. Diff, the difference between the local normalized (ln) values of the immunoprecipitated (ChIP) samples and the total (non-ChIP) DNA samples, for each of the ChIP samples, i.e., V (vegetative growth), F (onset of sporulation/fragmentation), S (midsporulation), and C (wild-type negative control, analyzed at the onset of sporulation/fragmentation). Adjusted *P* values (apv), significance values for each of the ChIP samples, i.e., V, F, S, and WT negative control (C) after adjusting for multiple testing by the Hochberg method as implemented in the *p.adjust* function of R. Peak (pk), each ChIP sample is qualified as being significant (*P* < E−04) by the identifier TRUE and insignificant (*P* > E−04) by the identifier FALSE. Left Gene, the identifier (SVEN no.) for the gene on the left of the identified ChIP peak. Right Gene, the identifier (SVEN no.) for the gene on the right of the identified ChIP peak. Distance, the distance (in bases) between the ChIP peak and the predicted start codon of the downstream gene. Start, the start position on the *S. venezuelae* genome (in bases) of the gene downstream of the ChIP peak. End, the end position on the *S. venezuelae* genome (in bases) of the gene downstream of the ChIP peak. Strand, the strand on which the gene is found (forward = 1; reverse = −1). Product, (possible) gene function based on annotation in StrepDB (http://strepdb.streptomyces.org.uk). Affy LogFC, the log fold change (log_2_ scale) in expression of the *whiA* mutant compared to wild-type *S. venezuelae* at the 10-, 12-, 14-, 16-, 18-, and 20-h time points. − indicates a decrease in expression of the gene in a *whiA* mutant compared to the wild type; + indicates an increase in expression of the gene in a *whiA* mutant compared to the wild type. Cells highlighted in red represent greater than a 2-fold increase in expression in the *whiA* mutant. Cells highlighted in yellow represent greater than a 2-fold decrease in expression in the *whiA* mutant. Table S1, XLSX file, 0.1 MB.

Table S2 Strains, plasmids, and oligonucleotide primers used in this study. Table S2, DOCX file, 0.1 MB.

## References

[1] Flärdh K Buttner MJ 2009 *Streptomyces* morphogenetics: dissecting differentiation in a filamentous bacterium. Nat. Rev. Microbiol. 7:36–49 19079351 10.1038/nrmicro1968

[2] McCormick JR 2009 Cell division is dispensable but not irrelevant in *Streptomyces*. Curr. Opin. Microbiol. 12:689–698 19889570 10.1016/j.mib.2009.10.004

[3] McCormick JR Flärdh K 2012 Signals and regulators that govern *Streptomyces* development. FEMS Microbiol. Rev. 36:206–231 22092088 10.1111/j.1574-6976.2011.00317.x PMC3285474

[4] Jakimowicz D van Wezel GP 2012 Cell division and DNA segregation in *Streptomyces*: how to build a septum in the middle of nowhere? Mol. Microbiol. 85:393–404 22646484 10.1111/j.1365-2958.2012.08107.x

[5] Aínsa JA Ryding NJ Hartley N Findlay KC Bruton CJ Chater KF 2000 WhiA, a protein of unknown function conserved among Gram-positive bacteria, is essential for sporulation in *Streptomyces coelicolor* A3. J. Bacteriol. 182:5470–5478 10986251 10.1128/jb.182.19.5470-5478.2000 PMC110991

[6] Flärdh K Findlay KC Chater KF 1999 Association of early sporulation genes with suggested developmental decision points in *Streptomyces coelicolor* A3. Microbiology 145:2229–2243 10517576 10.1099/00221287-145-9-2229

[7] Knizewski L Ginalski K 2007 Bacterial DUF.199/COG1481 proteins including sporulation regulator WhiA are distant homologs of LAGLIDADG homing endonucleases that retained only DNA binding. Cell Cycle 6:1666–1670 17603302 10.4161/cc.6.13.4471

[8] Kaiser BK Clifton MC Shen BW Stoddard BL 2009 The structure of a bacterial DUF199/WhiA protein: domestication of an invasive endonuclease. Structure 17:1368–1376 19836336 10.1016/j.str.2009.08.008 PMC2766575

[9] Kaiser BK Stoddard BL 2011 DNA recognition and transcriptional regulation by the WhiA sporulation factor. Sci. Rep. 1:156. 10.1038/srep00156 22355671 PMC3240954

[10] Taylor GK Stoddard BL 2012 Structural, functional and evolutionary relationships between homing endonucleases and proteins from their host organisms. Nucleic Acids Res. 40:5189–5200 22406833 10.1093/nar/gks226 PMC3384342

[11] Elliot MA Bibb MJ Buttner MJ Leskiw BK 2001 BldD is a direct regulator of key developmental genes in *Streptomyces coelicolor* A3(2). Mol. Microbiol. 40:257-269 11298292 10.1046/j.1365-2958.2001.02387.x

[12] Chater KF Chandra G 2008 The use of the rare UUA codon to define “expression space” for genes involved in secondary metabolism, development and environmental adaptation in *Streptomyces*. J. Microbiol. 46:1–11 18337685 10.1007/s12275-007-0233-1

[13] Chandra G Chater KF 2008 Evolutionary flux of potentially *bldA*-dependent *Streptomyces* genes containing the rare leucine codon TTA. Antonie Van Leeuwenhoek 94:111–126 18320344 10.1007/s10482-008-9231-5

[14] den Hengst CD Tran NT Bibb MJ Chandra G Leskiw BK Buttner MJ 2010 Genes essential for morphological development and antibiotic production in *Streptomyces coelicolor* are targets of BldD during vegetative growth. Mol. Microbiol. 78:361–379 20979333 10.1111/j.1365-2958.2010.07338.x

[15] Higo A Hara H Horinouchi S Ohnishi Y 2012 Genome-wide distribution of AdpA, a global regulator for secondary metabolism and morphological differentiation in *Streptomyces*, revealed the extent and complexity of the AdpA regulatory network. DNA Res. 19:259–273 22449632 10.1093/dnares/dss010 PMC3372375

[16] Bibb MJ Domonkos A Chandra G Buttner MJ 2012 Expression of the chaplin and rodlin hydrophobic sheath proteins in *Streptomyces venezuelae* is controlled by σ^BldN^ and a cognate anti-sigma factor, RsbN. Mol. Microbiol. 84:1033–1049 22582857 10.1111/j.1365-2958.2012.08070.x

[17] Glazebrook MA Doull JL Stuttard C Vining LC 1990 Sporulation of *Streptomyces venezuelae* in submerged cultures. J. Gen. Microbiol. 136:581–588 2391493 10.1099/00221287-136-3-581

[18] Gust B Challis GL Fowler K Kieser T Chater KF 2003 PCR-targeted *Streptomyces* gene replacement identifies a protein domain needed for biosynthesis of the sesquiterpene soil odor geosmin. Proc. Natl. Acad. Sci. U. S. A. 100:1541–1546 12563033 10.1073/pnas.0337542100 PMC149868

[19] Gust B Chandra G Jakimowicz D Yuqing T Bruton CJ Chater KF 2004 Lambda red-mediated genetic manipulation of antibiotic-producing *Streptomyces*. Adv. Appl. Microbiol. 54:107–128 15251278 10.1016/S0065-2164(04)54004-2

[20] Teytelman L Ozaydin B Zill O Lefrançois P Snyder M Rine J Eisen MB 2009 Impact of chromatin structures on DNA processing for genomic analyses. PLoS One 4:e6700. 10.1371/journal.pone.0006700 19693276 PMC2725323

[21] Bailey TL Elkan C 1994 Fitting a mixture model by expectation maximization to discover motifs in biopolymers, p 28–36 In Altman R Brutlag D Karp P Lathrop R Searls D (ed), Proceedings of the Second International Conference on Intelligent Systems for Molecular Biology. AAAI Press, Menlo Park, CA 7584402

[22] Jakimowicz D Mouz S Zakrzewska-Czerwinska J Chater KF 2006 Developmental control of a *parAB* promoter leads to formation of sporulation-associated ParB complexes in *Streptomyces coelicolor*. J. Bacteriol. 188:1710–1720 16484182 10.1128/JB.188.5.1710-1720.2006 PMC1426544

[23] Hempel AM Cantlay S Molle V Wang SB Naldrett MJ Parker JL Richards DM Jung YG Buttner MJ Flärdh K 2012 The Ser/Thr protein kinase AfsK regulates polar growth and hyphal branching in the filamentous bacteria *Streptomyces*. Proc. Natl. Acad. Sci. U. S. A. 109:E2371–E2379 22869733 10.1073/pnas.1207409109 PMC3435184

[24] Flärdh K Richards DM Hempel AM Howard M Buttner MJ 2012 Regulation of apical growth and hyphal branching in *Streptomyces*. Curr. Opin. Microbiol. 15:737–743 23153774 10.1016/j.mib.2012.10.012

[25] Fuchino K Bagchi S Cantlay S Sandblad L Wu D Bergman J Kamali-Moghaddam M Flärdh K Ausmees N 2013 Dynamic gradients of an intermediate filament-like cytoskeleton are recruited by a polarity landmark during apical growth. Proc. Natl. Acad. Sci. U. S. A. 110:E1889–E1897 23641002 10.1073/pnas.1305358110 PMC3666699

[26] Holmes NA Walshaw J Leggett RM Thibessard A Dalton KA Gillespie MD Hemmings AM Gust B Kelemen GH 2013 Coiled-coil protein Scy is a key component of a multiprotein assembly controlling polarized growth in *Streptomyces*. Proc. Natl. Acad. Sci. U. S. A. 110:E397–E406 23297235 10.1073/pnas.1210657110 PMC3562780

[27] Bagchi S Tomenius H Belova LM Ausmees N 2008 Intermediate filament-like proteins in bacteria and a cytoskeletal function in *Streptomyces*. Mol. Microbiol. 70:1037–1050 18976278 10.1111/j.1365-2958.2008.06473.x PMC2680258

[28] Flärdh K 2003 Essential role of DivIVA in polar growth and morphogenesis in *Streptomyces coelicolor* A3(2). Mol. Microbiol. 49:1523-1536 12950918 10.1046/j.1365-2958.2003.03660.x

[29] Flärdh K 2003 Growth polarity and cell division in *Streptomyces*. Curr. Opin. Microbiol. 6:564–571 14662351 10.1016/j.mib.2003.10.011

[30] Ditkowski B Holmes N Rydzak J Donczew M Bezulska M Ginda K Kedzierski P Zakrzewska-Czerwinska J Kelemen GH Jakimowicz D 2013 Dynamic interplay of ParA with the polarity protein, Scy, coordinates the growth with chromosome segregation in *Streptomyces coelicolor*. Open Biol. 3:130006. 10.1098/rsob.130006 23536551 PMC3718342

[31] de Jong W Wösten HA Dijkhuizen L Claessen D 2009 Attachment of *Streptomyces coelicolor* is mediated by amyloidal fimbriae that are anchored to the cell surface via cellulose. Mol. Microbiol. 73:1128–1140 19682261 10.1111/j.1365-2958.2009.06838.x

[32] Xu H Chater KF Deng Z Tao M 2008 A cellulose synthase-like protein involved in hyphal tip growth and morphological differentiation in *Streptomyces*. J. Bacteriol. 190:4971–4978 18487344 10.1128/JB.01849-07 PMC2446991

[33] Liman R Facey PD van Keulen G Dyson PJ Del Sol R 2013 A laterally acquired galactose oxidase-like gene is required for aerial development during osmotic stress in *Streptomyces coelicolor*. PLoS One 8:e54112. 10.1371/journal.pone.0054112 23326581 PMC3543389

[34] Flärdh K Leibovitz E Buttner MJ Chater KF 2000 Generation of a non-sporulating strain of *Streptomyces coelicolor* A3(2) by the manipulation of a developmentally controlled *ftsZ* promoter. Mol. Microbiol. 38:737–749 11115109 10.1046/j.1365-2958.2000.02177.x

[35] Willemse J Mommaas AM van Wezel GP 2012 Constitutive expression of *ftsZ* overrides the *whi* developmental genes to initiate sporulation of *Streptomyces coelicolor*. Antonie Van Leeuwenhoek 101:619–632 22113698 10.1007/s10482-011-9678-7 PMC3278627

[36] Mercer KL Weiss DS 2002 The *Escherichia coli* cell division protein FtsW is required to recruit its cognate transpeptidase, FtsI (PBP3), to the division site. J. Bacteriol. 184:904–912 11807049 10.1128/jb.184.4.904-912.2002 PMC134820

[37] Mohammadi T van Dam V Sijbrandi R Vernet T Zapun A Bouhss A Diepeveen-de Bruin M Nguyen-Distèche M de Kruijff B Breukink E 2011 Identification of FtsW as a transporter of lipid-linked cell wall precursors across the membrane. EMBO J. 30:1425–1432 21386816 10.1038/emboj.2011.61 PMC3102273

[38] Mistry BV Del Sol R Wright C Findlay K Dyson P 2008 FtsW is a dispensable cell division protein required for Z-ring stabilization during sporulation septation in *Streptomyces coelicolor*. J. Bacteriol. 190:5555–5566 18556789 10.1128/JB.00398-08 PMC2519378

[39] Bennett JA Yarnall J Cadwallader AB Kuennen R Bidey P Stadelmaier B McCormick JR 2009 Medium-dependent phenotypes of *Streptomyces coelicolor* with mutations in *ftsI* or *ftsW*. J. Bacteriol. 191:661–664 18978049 10.1128/JB.01048-08 PMC2620802

[40] McCormick JR Su EP Driks A Losick R 1994 Growth and viability of *Streptomyces coelicolor* mutant for the cell division gene *ftsZ*. Mol. Microbiol. 14:243–254 7830569 10.1111/j.1365-2958.1994.tb01285.x

[41] Wang L Yu Y He X Zhou X Deng Z Chater KF Tao M 2007 Role of an FtsK-like protein in genetic stability in *Streptomyces coelicolor* A3(2). J. Bacteriol. 189:2310-2318 17209017 10.1128/JB.01660-06 PMC1899397

[42] Arends SJ Kustusch RJ Weiss DS 2009 ATP-binding site lesions in FtsE impair cell division. J. Bacteriol. 191:3772–3784 19376877 10.1128/JB.00179-09 PMC2698383

[43] Schmidt KL Peterson ND Kustusch RJ Wissel MC Graham B Phillips GJ Weiss DS 2004 A predicted ABC transporter, FtsEX, is needed for cell division in *Escherichia coli*. J. Bacteriol. 186:785–793 14729705 10.1128/JB.186.3.785-793.2004 PMC321481

[44] de Leeuw E Graham B Phillips GJ ten Hagen-Jongman CM Oudega B Luirink J 1999 Molecular characterization of *Escherichia coli* FtsE and FtsX. Mol. Microbiol. 31:983–993 10048040 10.1046/j.1365-2958.1999.01245.x

[45] Corbin BD Wang Y Beuria TK Margolin W 2007 Interaction between cell division proteins FtsE and FtsZ. J. Bacteriol. 189:3026–3035 17307852 10.1128/JB.01581-06 PMC1855847

[46] Jakimowicz D Chater K Zakrzewska-Czerwínska J 2002 The ParB protein of *Streptomyces coelicolor* A3(2) recognizes a cluster of *parS* sequences within the origin-proximal region of the linear chromosome. Mol. Microbiol. 45:1365–1377 12207703 10.1046/j.1365-2958.2002.03102.x

[47] Jakimowicz D Gust B Zakrzewska-Czerwinska J Chater KF 2005 Developmental-stage-specific assembly of ParB complexes in *Streptomyces coelicolor* hyphae. J. Bacteriol. 187:3572–3580 15866947 10.1128/JB.187.10.3572-3580.2005 PMC1112017

[48] Jakimowicz D Zydek P Kois A Zakrzewska-Czerwinska J Chater KF 2007 Alignment of multiple chromosomes along helical ParA scaffolding in sporulating *Streptomyces* hyphae. Mol. Microbiol. 65:625–641 17635186 10.1111/j.1365-2958.2007.05815.x

[49] Ishikawa S Kawai Y Hiramatsu K Kuwano M Ogasawara N 2006 A new FtsZ-interacting protein, YlmF, complements the activity of FtsA during progression of cell division in *Bacillus subtilis*. Mol. Microbiol. 60:1364–1380 16796675 10.1111/j.1365-2958.2006.05184.x

[50] Hamoen LW Meile JC de Jong W Noirot P Errington J 2006 SepF, a novel FtsZ-interacting protein required for a late step in cell division. Mol. Microbiol. 59:989–999 16420366 10.1111/j.1365-2958.2005.04987.x

[51] Tran NT Den Hengst CD Gomez-Escribano JP Buttner MJ 2011 Identification and characterization of CdgB, a diguanylate cyclase involved in developmental processes in *Streptomyces coelicolor*. J. Bacteriol. 193:3100–3108 21515767 10.1128/JB.01460-10 PMC3133206

[52] Hull TD Ryu MH Sullivan MJ Johnson RC Klena NT Geiger RM Gomelsky M Bennett JA 2012 Cyclic di-GMP phosphodiesterases RmdA and RmdB are involved in regulating colony morphology and development in *Streptomyces coelicolor*. J. Bacteriol. 194:4642–4651 22753061 10.1128/JB.00157-12 PMC3415515

[53] Ryding NJ Kelemen GH Whatling CA Flärdh K Buttner MJ Chater KF 1998 A developmentally regulated gene encoding a repressor-like protein is essential for sporulation in *Streptomyces coelicolor* A3. Mol. Microbiol. 29:343–357 9701826 10.1046/j.1365-2958.1998.00939.x

[54] Aínsa JA Parry HD Chater KF 1999 A response regulator-like protein that functions at an intermediate stage of sporulation in *Streptomyces coelicolor* A3. Mol. Microbiol. 34:607–619 10564501 10.1046/j.1365-2958.1999.01630.x

[55] Borovok I Gorovitz B Yanku M Schreiber R Gust B Chater K Aharonowitz Y Cohen G 2004 Alternative oxygen-dependent and oxygen-independent ribonucleotide reductases in *Streptomyces*: cross-regulation and physiological role in response to oxygen limitation. Mol. Microbiol. 54:1022–1035 15522084 10.1111/j.1365-2958.2004.04325.x

[56] Grinberg I Shteinberg T Gorovitz B Aharonowitz Y Cohen G Borovok I 2006 The *Streptomyces* NrdR transcriptional regulator is a Zn ribbon/ATP cone protein that binds to the promoter regions of class Ia and class II ribonucleotide reductase operons. J. Bacteriol. 188:7635–7644 16950922 10.1128/JB.00903-06 PMC1636249

[57] Grinberg I Shteinberg T Hassan AQ Aharonowitz Y Borovok I Cohen G 2009 Functional analysis of the *Streptomyces coelicolor* NrdR ATP-cone domain: role in nucleotide binding, oligomerization, and DNA interactions. J. Bacteriol. 191:1169–1179 19047342 10.1128/JB.01145-08 PMC2632000

[58] Herrick J Sclavi B 2007 Ribonucleotide reductase and the regulation of DNA replication: an old story and an ancient heritage. Mol. Microbiol. 63:22–34 17229208 10.1111/j.1365-2958.2006.05493.x

[59] Ruban-Ośmiałowska B Jakimowicz D Smulczyk-Krawczyszyn A Chater KF Zakrzewska-Czerwinska J 2006 Replisome localization in vegetative and aerial hyphae of *Streptomyces coelicolor*. J. Bacteriol. 188:7311–7316 17015671 10.1128/JB.00940-06 PMC1636232

[60] Turnbough CL Switzer RL 2008 Regulation of pyrimidine biosynthetic gene expression in bacteria: repression without repressors. Microbiol. Mol. Biol. Rev. 72:266–300 18535147 10.1128/MMBR.00001-08 PMC2415746

[61] Ingerson-Mahar M Gitai Z 2012 A growing family: the expanding universe of the bacterial cytoskeleton. FEMS Microbiol. Rev. 36:256–266 22092065 10.1111/j.1574-6976.2011.00316.x PMC4114309

[62] Barry RM Gitai Z 2011 Self-assembling enzymes and the origins of the cytoskeleton. Curr. Opin. Microbiol. 14:704–711 22014508 10.1016/j.mib.2011.09.015 PMC3234109

[63] Ingerson-Mahar M Briegel A Werner JN Jensen GJ Gitai Z 2010 The metabolic enzyme CTP synthase forms cytoskeletal filaments. Nat. Cell Biol. 12:739–746 20639870 10.1038/ncb2087 PMC3210567

[64] Gao C Hindra Mulder D Yin C Elliot MA 2012 Crp is a global regulator of antibiotic production in *Streptomyces*. mBio 3(6):e00407-12. 10.1128/mBio.00407-12 23232715 PMC3520106

[65] Zhang G Tian Y Hu K Feng C Tan H 2010 SCO3900, co-transcripted with three downstream genes, is involved in the differentiation of *Streptomyces coelicolor*. Curr. Microbiol. 60:268–273 20012957 10.1007/s00284-009-9536-2

[66] Dalton KA Thibessard A Hunter JI Kelemen GH 2007 A novel compartment, the “subapical stem” of the aerial hyphae, is the location of a *sigN*-dependent, developmentally distinct transcription in *Streptomyces coelicolor*. Mol. Microbiol. 64:719–737 17462019 10.1111/j.1365-2958.2007.05684.x

[67] Molle V Palframan WJ Findlay KC Buttner MJ 2000 WhiD and WhiB, homologous proteins required for different stages of sporulation in *Streptomyces coelicolor* A3. J. Bacteriol. 182:1286–1295 10671449 10.1128/jb.182.5.1286-1295.2000 PMC94414

[68] den Hengst CD Buttner MJ 2008 Redox control in Actinobacteria. Biochim. Biophys. Acta 1780:1201–1216 18252205 10.1016/j.bbagen.2008.01.008

[69] Gao B Paramanathan R Gupta RS 2006 Signature proteins that are distinctive characteristics of Actinobacteria and their subgroups. Antonie Van Leeuwenhoek 90:69–91 16670965 10.1007/s10482-006-9061-2

[70] Jakimowicz P Cheesman MR Bishai WR Chater KF Thomson AJ Buttner MJ 2005 Evidence that the *Streptomyces* developmental protein WhiD, a member of the WhiB family, binds a [4Fe-4S] cluster. J. Biol. Chem. 280:8309–8315 15615709 10.1074/jbc.M412622200

[71] Singh A Guidry L Narasimhulu KV Mai D Trombley J Redding KE Giles GI Lancaster JR Steyn AJ 2007 *Mycobacterium tuberculosis* WhiB3 responds to O_2_ and nitric oxide via its [4Fe-4S] cluster and is essential for nutrient starvation survival. Proc. Natl. Acad. Sci. U. S. A. 104:11562–11567 17609386 10.1073/pnas.0700490104 PMC1906726

[72] Crack JC Smith LJ Stapleton MR Peck J Watmough NJ Buttner MJ Buxton RS Green J Oganesyan VS Thomson AJ Le Brun NE 2011 Mechanistic insight into the nitrosylation of the [4Fe-4S] cluster of WhiB-like proteins. J. Am. Chem. Soc. 133:1112–1121 21182249 10.1021/ja109581t PMC3117330

[73] Smith LJ Stapleton MR Fullstone GJ Crack JC Thomson AJ Le Brun NE Hunt DM Harvey E Adinolfi S Buxton RS Green J 2010 *Mycobacterium tuberculosis* WhiB1 is an essential DNA-binding protein with a nitric oxide-sensitive iron-sulfur cluster. Biochem. J. 432:417–427 20929442 10.1042/BJ20101440 PMC2992795

[74] Chandra G Chater KF Bornemann S 2011 Unexpected and widespread connections between bacterial glycogen and trehalose metabolism. Microbiology 157:1565–1572 21474533 10.1099/mic.0.044263-0

[75] Bruton CJ Plaskitt KA Chater KF 1995 Tissue-specific glycogen branching isoenzymes in a multicellular prokaryote, *Streptomyces coelicolor* A3. Mol. Microbiol. 18:89–99 8596463 10.1111/j.1365-2958.1995.mmi_18010089.x

[76] Yeo M Chater K 2005 The interplay of glycogen metabolism and differentiation provides an insight into the developmental biology of *Streptomyces coelicolor*. Microbiology 151:855–861 15758231 10.1099/mic.0.27428-0

[77] Rueda B Miguélez EM Hardisson C Manzanal MB 2001 Changes in glycogen and trehalose content of *Streptomyces brasiliensis* hyphae during growth in liquid cultures under sporulating and non-sporulating conditions. FEMS Microbiol. Lett. 194:181–185 11164305 10.1111/j.1574-6968.2001.tb09466.x

[78] Vasilyev NN Kutlubaeva ZS Ugarov VI Chetverina HV Chetverin AB 2013 Ribosomal protein S1 functions as a termination factor in RNA synthesis by Qβ phage replicase. Nat. Commun. 4:1781. 10.1038/ncomms2807 23653193

[79] Greive SJ Lins AF von Hippel PH 2005 Assembly of an RNA-protein complex. Binding of NusB and NusE (S10) proteins to boxA RNA nucleates the formation of the antitermination complex involved in controlling rRNA transcription in *Escherichia coli*. J. Biol. Chem. 280:36397–36408 16109710 10.1074/jbc.M507146200

[80] Adams DW Errington J 2009 Bacterial cell division: assembly, maintenance and disassembly of the Z ring. Nat. Rev. Microbiol. 7:642–653 19680248 10.1038/nrmicro2198

[81] Willemse J Borst JW de Waal E Bisseling T van Wezel GP 2011 Positive control of cell division: FtsZ is recruited by SsgB during sporulation of *Streptomyces*. Genes Dev. 25:89–99 21205868 10.1101/gad.600211 PMC3012939

[82] Görke B Foulquier E Galinier A 2005 YvcK of *Bacillus subtilis* is required for a normal cell shape and for growth on Krebs cycle intermediates and substrates of the pentose phosphate pathway. Microbiology 151:3777–3791 16272399 10.1099/mic.0.28172-0

[83] Datsenko KA Wanner BL 2000 One-step inactivation of chromosomal genes in *Escherichia coli* K-12 using PCR products. Proc. Natl. Acad. Sci. U. S. A. 97:6640–6645 10829079 10.1073/pnas.120163297 PMC18686

[84] Paget MSB Chamberlin L Atrih A Foster SJ Buttner MJ 1999 Evidence that the extracytoplasmic function sigma factor, σ^E^, is required for normal cell wall structure in *Streptomyces coelicolor* A3(2). J. Bacteriol. 181:204–211 9864331 10.1128/jb.181.1.204-211.1999 PMC103550

[85] Stuttard C 1982 Temperate phages of *Streptomyces venezuelae*: lysogeny and host specificity shown by phages SV1 and SV2. Microbiology 128:115–121

[86] Kieser T Bibb MJ Buttner MJ Chater KF Hopwood DA 2000 Practical Streptomyces genetics. John Innes Foundation, Norwich, United Kingdom

[87] Gregory MA Till R Smith MC 2003 Integration site for *Streptomyces* phage ΦBT1 and development of site-specific integrating vectors. J. Bacteriol. 185:5320–5323 12923110 10.1128/JB.185.17.5320-5323.2003 PMC180994

[88] Hesketh A Kock H Mootien S Bibb M 2009 The role of *absC*, a novel regulatory gene for secondary metabolism, in zinc-dependent antibiotic production in *Streptomyces coelicolor* A3. Mol. Microbiol. 74:1427–1444 19906184 10.1111/j.1365-2958.2009.06941.x

[89] Core R Team 2013 R: a language and environment for statistical computing. R Foundation for Statistical Computing, Vienna, Austria http://www.R-project.org/

[90] Gautier L Cope L Bolstad BM Irizarry RA 2004 Affy-analysis of Affymetrix GeneChip data at the probe level. Bioinformatics 20:307–315 14960456 10.1093/bioinformatics/btg405

[91] Smyth GK 2005 Limma: linear models for microarray data, p 397–420 In Gentleman R Carey V Dudoit S Irizarry R Huber W (ed), Bioinformatics and computational biology solutions using R and Bioconductor. Springer Verlag, New York, NY.

[92] Langmead B Salzberg SL 2012 Fast gapped-read alignment with Bowtie 2. Nat. Methods 9:357–359 22388286 10.1038/nmeth.1923 PMC3322381

[93] Stajich JE Block D Boulez K Brenner SE Chervitz SA Dagdigian C Fuellen G Gilbert JG Korf I Lapp H Lehväslaiho H Matsalla C Mungall CJ Osborne BI Pocock MR Schattner P Senger M Stein LD Stupka E Wilkinson MD Birney E 2002 The Bioperl toolkit: Perl modules for the life sciences. Genome Res. 12:1611–1618 12368254 10.1101/gr.361602 PMC187536

[94] Nicol JW Helt GA Blanchard SG Raja A Loraine AE 2009 The integrated genome browser: free software for distribution and exploration of genome-scale datasets. Bioinformatics 25:2730–2731 19654113 10.1093/bioinformatics/btp472 PMC2759552

