# Supplementary material for: Genes Required for Aerial Growth, Cell Division, and Chromosome Segregation Are Targets of WhiA before Sporulation in Streptomyces venezuelae
Source: mBio. 2013 Sep 24;4(5):e00684-13. doi: 10.1128/mBio.00684-13 (PMC3781837; doi:10.1128/mBio.00684-13)
Supplement: Text S1 — Supplemental materials and methods. Download [file mbo005131625s1.docx]

**Western Blotting.** Samples of frozen mycelium, originating from 5 ml liquid MYM cultures, were resuspended in 0.4 ml ice-cold sonication buffer [20 mM Tris pH 8.0, 5 mM EDTA, 1 x EDTA-free protease inhibitors (Roche)] and sonicated (5x 15 sec on/15 sec off) at 4.5 micron amplitude. Lysates were then centrifuged at 16,000 xg for 15 min at 4˚C to remove cell debris. Total protein concentration was determined using the Bradford assay (Biorad). Equal amounts of total protein from each sample were loaded on a 12.5% polyacrylamide SDS-PAGE gel. After electrophoresis, transfer was carried out to a Hybond-C Extra nylon membrane (Amerhsam Pharmacia Biotech) using the Invitrogen XCell II Blot system. For detection of WhiA, polyclonal antibody raised in rabbit was used (Cambridge Research Biochemicals). For detection of 3xFLAG-[Gly_4_Ser]_3_-WhiA, anti-FLAG antibody (Sigma F4725) was used. In both cases the primary antibody was diluted in a ratio of 1:2500. WhiA and 3xFLAG-[Gly_4_Ser]_3_-WhiA was visualised via an anti–rabbit IgG alkaline phosphatase secondary antibody (sigma A8025), diluted 1:5000 and detected directly on the membrane using the SigmaFast system (Sigma) that uses BCIP/NBT (5-Bromo-4-chloro-3-indolyl phosphate/Nitro blue tetrazolium) as a substrate.

**DNase I footprinting.** DNase I footprinting experiments were carried out essentially as described previously (14) and according to the description supplied with the Sure Track footprinting kit (Amersham Pharmacia Biotech). DNA fragments were prepared by PCR with combinations of primers *whiA*_fwd and whiA_rev, *nrdR*_fwd and *nrdR*_rev, *ftsW­*_fwd and *ftsW*_rev, *filP*_fwd and *filP*_rev, *cslA*_fwd and *cslA*_rev, *sven1406*_fwd and *sven1406*_rev, *sven4724/5*_fwd and *sven4724/5*_rev, *treZ*_fwd and *treZ*_rev, *sven3535*_fwd and *sven3535*_rev, *sven3229*_fwd and *sven3229*_rev (Table S2), to generate DNA probes overlapping potential WhiA binding sites near the genes *whiA*, *nrdR*, *ftsW*, *cslA*, *sven1406*, *sven4724*, *sven5890*, *sven3535* and *sven3229* respectively. Oligonucleotides were first end-labelled with T4 polynucleotide kinase (Amersham Pharmacia Biotech) and [γ-^32^P]-ATP as described by the manufacturer. Binding reactions were carried out at room temperature for 30 min in 1 x TAP buffer (50 mM Tris-acetate pH 7.9, 100 mM Potassium-acetate, 8 mM magnesium acetate, 27 mM ammonium acetate, 1 mM DTT, 3.5% PEG6000) in a total volume of 40 µl, and in the presence of approximately 150 000 cpm of the DNA probe. Following incubation, 10 μl containing 3 units of DNase I (Promega) and 1 μl of CaCl_2_ was added, mixed and incubated for 1 min. The reaction was stopped by addition of 140 μl stop solution [192 mM NaAc, 32 mM EDTA, 0.14% SDS, 70 μg yeast-tRNA (Invitrogen)]. Samples were then phenol-chloroform extracted prior to ethanol (96%) precipitation. The pellet was vacuum-dried and resuspended in 5 μl of formamide loading dye (95% formamide, 20 mM EDTA pH 8.0, 0.1% bromophenol blue, 0.1% xyelene cyanol FF). 2.5 μl of each sample was loaded on a 6% sequencing gel, next to a G+A ladder, prepared according to the Sure Track footprinting kit (Amersham Pharmacia Biotech). The gel was then vacuum-dried before imaging using image plates, visualised using the FUJIFILM FLA-7000.

**Light microscopy**. Coverslips were placed gently on the surface of *S. venezuelae* colonies grown on solid MYM medium for 4 days and then lifted and analysed by phase-contrast light microscopy with oil immersion (Carl Zeiss; 100/2.0 objective). Alternatively, the coverslip was embedded at an angle in the agar and the strain inoculated along the inside edge of the coverslip.

**Scanning electron microscopy**. Colonies were mounted on the surface of an aluminum stub with optimal cutting temperature compound (Agar Scientific Ltd, Essex, UK), plunged into liquid nitrogen slush at approximately -210°C to cryopreserve the material, and transferred to the cryostage of an Alto 2500 cryotransfer system (Gatan, Oxford, England) attached to a Zeiss Supra 55 VP field emission gun scanning electron microscope (Carl Zeiss Ltd, Germany). The surface frost was sublimated at -95°C for 3 min before the sample was sputter coated with platinum for 2 min at 10 mA at below -110°C. Finally, the sample was moved onto the cryostage in the main chamber of the microscope, held at approximately -130°C, and viewed at 1.2 to 5.0 kV.

**Transmission electron microscopy.** Colonies were fixed in 2.5% (vol/vol) glutaraldehyde in 0.05 M sodium cacodylate, stained with osmic acid, and embedded in LR White resin according to the manufacturer’s instructions (The London Resin Co.). Sections were cut on a UC6 ultramicrotome (Leica Microsystems (UK) Ltd, Milton Keynes) and examined in a JEOL 1200 EX transmission electron microscope (Welwyn Garden City, UK).

**Fluorescence microscopy.** DAPI (4',6-diamidino-2-phenylindole) solutions in water were used to stain DNA at 10 µg/ml. A 2mg/ml stock of Nile Red in DMSO was diluted to final concentration of 10 µg/ml and used to stain membranes. Approximately 20 μl of the stain was gently dropped onto the sample which had been growing on the surface of a cover slip for 1-2 days at 30 ˚C. After 2 to 3 minutes incubation in the dark, the coverslip was gently rinsed 6 times in water and studied using a Cairns CCD microscope at x100 magnification. Digital images were assembled using Image J and ADOBE photoshop software.
