# Supplementary material for: Genes Required for Aerial Growth, Cell Division, and Chromosome Segregation Are Targets of WhiA before Sporulation in Streptomyces venezuelae
Source: mBio. 2013 Sep 24;4(5):e00684-13. doi: 10.1128/mBio.00684-13 (PMC3781837; doi:10.1128/mBio.00684-13)
Supplement: Table S2 — Strains, plasmids, and oligonucleotide primers used in this study. [file mbo005131625st2.docx]

Table S2 Strains, Plasmids and Oligonucleotide primers used in this study

**Strains Relevant genotype/comments Source/reference**

*S.venezuelae*

ATCC10712 Wild-type

SV11 Δ*whiA::apr* This study

SV11-pIJ6760 Δ*whiA::apr* with pIJ6760 integrated at the ΦBT1 attachment site This study

SV11-pIJ10600 Δ*whiA::apr* with pIJ10600 integrated at the ΦBT1 attachment site This study

SV11-pIJ10601 Δ*whiA::apr* with pIJ10601 integrated at the ΦBT1 attachment site This study

*E.coli*

ET12567(pUZ8002) ET12567 containing helper plasmid pUZ8002 Paget et al., (1999)

BW25113 Δ(*araD*-*araB*)*567* Δ *lacZ4787*(::*rrnB-4*) *lacIp-4000*(*lacI^Q^*), l*-rpoS369*(*Am*) *rph-1* Datsenko and Wanner (2000)

Δ (*rhaD*-*rhaB*)*568 hsdR514*

**Plasmids**

pIJ773 Plasmid template for amplification of the *apr oriT* cassette for ‘Redirect’ PCR-targeting Gust *et al*. (2003)

pIJ790 Modified l RED recombination plasmid [*oriR101*] [*repA101*(ts)] *araBp-gam-be-exo* Gust *et al*. (2003)

pMS82 Plasmid cloning vector for the conjugal transfer of DNA from *E. coli* to *Streptomyces* spp. Gregory *et al*. (2003)

Integrates site specifically at the ΦBT1 attachment site (Hyg^R^)

pIJ6760 pMS82 carrying *whiA* driven from its own promoter This study

pIJ10600 pMS82 carrying sequence encoding 3xFLAG-WhiA driven from its own promoter This study

pIJ10601 pMS82 carrying sequence encoding 3xFLAG-[Gly_4_Ser]_3_-WhiA driven from its own promoter This study

**Primers** **Sequence**

whiAdisfor cgaccgtatttttcggatgcatggaaggatcggcccatgattccggggatccgtcgacc

whiAdisrev gcggagttcgtcaattccgtccggggacggctccggtcatgtaggctggagctgcttc

whiAconfor gctccgcgaagcatgacc

whiAconrev gagtgccacgagcgtacg

whiAcompfor cggaagcttcggagaaaccgagggcttc

whiAcomprev cggaagctttccggggacggctccggtc

whiAFLAGextfor gaactgctggacgcgctg

whiAFLAGextrev tgggcctgtcaagggcg

whiAFLAGfusrev tcgatgtcgtggtccttgtagtcgccgtcgtggtccttgtagtccatgggccgatccttcca

whiAFLAGfusfor cgactacaaggaccacgacatcgactacaaggacgatgacgacaagatggcgatgacggcagcg

whiAFLAGnesfor ggcgaagcttcggagaaaccgagggcttc

whiAFLAGnesrev ggggtacctccggggacggctccggtc

whiAlinkfusrev cgccagagccacctccgcctgaaccgcctccacccttgtcgtcatcgtcctt

whiAlinkfusfor gttcaggcggaggtggctctggcggtggcggtagtatggcgatgacggcagcg

*whiA*_fwd gcgccgcagcccggagaa

*whiA*_rev gcggcaccgagccgcttg

*nrdR*_fwd agcatccggtgcaccctc

*nrdR*_rev ccgatgccatccaaccac

*ftsW*_fwd gaccgcctcggacatcg

*ftsW*_rev gaactggtggagaagtcgg

*filP*_fwd ccggaattcccactcaaaaggctggac

*filP*_rev cgcggatcctaaccgcgccgcacgagc

*cslA*_fwd gacatacccactcggact

*cslA*_rev gtgtcgtagtcgtagcct

*treZ*_fwd gacgtgccgcgcatccg

*treZ*_rev gcccacacctcgaagagc

*sven1406*_fwd gtcgtcttacctccttgg

*sven1406*_rev ggatgcatgcctgcgcac

*sven4724/5*_fwd gctgctccactccaccaac

*sven4724/5*_rev cgacgccgaccttcggtc

*sven3535*_fwd gcgccgttggggccaag

*sven3535*_rev ggcggtacgggagcggc

*sven3229*_fwd gtctcgcgcatgtgaggg

*sven3229*_rev gatggcgagggcacggc
